# Spirit of the Foreign Periodicals

**Published:** 1834-01-01

**Authors:** 


### Spirit of the Foreign Periodicals, &c.

### I. Use of Acetas Plumbi in several Pulmonary Affections.

*Case* 1. A woman, aged 32, of a phthisical constitution, was labouring under the symptoms of general pyrexia, accompanied with frequent cough and purulent bloody expectoration. She had suffered a smart attack of pleuritis twelve months before, and from that time had become considerably emaciated. A small bleeding and the employment of sal ammoniac, with small doses of the tart. antimon. and an occasional powder of calomel, relieved the fever and dyspnœa; the sputa were now free of any blood, but became more and more purulent. I ordered her the acetas plumbi and opium (of each ¼ gr.) every eight hours, and in the course of eight days she was astonishingly improved. Under the use of a decoction of lichen and polygala amara (boiled together till a complete jelly is formed), she quite recovered her health.

*Case* 2. A man, aged 33, who had suffered repeatedly from attacks of pneumonia, was again labouring under its symptoms; they had lasted for seven days, when Dr. R. was called. By large bleedings and the use of nitre, combined with tart. antimon. in aqua laurocerasi, the inflammation was speedily arrested; but there remained a most copious expectoration, and the sputa were assuming a more purulent appearance. Pills, composed of the acetas plumbi and opium, were given with very marked benefit; the use of them was continued for six weeks, after which time the patient was entirely well.

*Case* 3. A child, five years old, had been treated by many different physicians for a phthisical irritation of the lungs, with repeated blisters, leeches, and the use of digitalis. The little patient expectorated a vast quantity of sputa, when I ordered him the following—

℞. Sacchari saturni.. gr. ij.

Infus. digitalis . . . unc. vj.

Laudani liquidi .. ℈j. Misce.

Capt. ʒij ad ʒiv. 6tâ vel 4tâ quâque horâ.

In three days the expectoration was greatly diminished, and the boy improved in other respects. He speedily was quite well.

*Case* 4. May 16th. B. W. aged 44, a professional musician, after exposure to cold, was seized with shiverings, followed by heat, with severe pain in the chest, laborious respiration, cough, and frothy discoloured expectoration. Venesection to a pint, and repeated doses of nitre and antimony ordered.

16th. All the symptoms aggravated; blood exhibiting a thick buffy coat—venesection to be repeated. While the blood was flowing, he felt himself much relieved; but immediately afterwards all his distress returned; the frothy sputa were in enormous quantities, so that the patient could with difficulty expectorate—the mucous rattle was exceedingly loud, and the breathing was much oppressed. A grain of calomel, and three of the red sulphuret of antimony, were ordered to be given every two hours, and the refrigerant mixture to be continued.

Little or no relief, however, was procured; the gurgling and rattling in the chest were truly frightful—the sputa were still frothy and tinged with pure blood, and their expectoration was painful and distressing. The bleeding from the arm was repeated once more, in consequence of the blood having presented, on both occasions, a very thick and tough crust; but no advantage followed, and being now alarmed that the accumulation of the sputa in the air-cells, and that the co-existing infiltration of the substance of the lungs, might speedily suffocate the patient, I resolved to commence the use of the acetas plumbi and opium. Three grains of the salt were dissolved in six ozs. of cherry-laurel water, and half a drachm of laudanum added; a table-spoonful every three hours. In the evening the symptoms were already much relieved, the expectoration less, and more easy, and the pulse reduced to 90 beats. Occasional delirium occurred; but having observed frequently, in many formidable cases, that this symptom appeared on the supervention of a critical change, I was rather pleased than distressed at its occurrence.

17th. Amendment has gone on progressively; delirium less frequent and continued—pectoral symptoms much more easy—breathing almost natural—pulse 75—skin perspiring comfortably.

The medicine being now discontinued for two days, a relapse of all the distress returned, cough, dyspnœa, difficult expectoration, and great anxiety; fortunately, by immediately resuming the medicine as before, the symptoms were once more subdued, and the cure was completed under the use of a jelly, prepared of lichen and the polygala amara.

*Case* 5. A woman, aged 58, had laboured under an inflammatory affection of the chest for eight days, when Dr. R. was summoned to her assistance. She had alternate chills and heats, pain in the side, short distressing cough, very scanty expectoration, and great anxiety. The pulse was 80, soft and weak. The pain and feverish symptoms were relieved by bleeding and the use of digitalis, with nitre. On the following day there was a return of her distress, and recourse was, therefore, had again to bloodletting; the blood at both times was strongly buffed. But although the stitch in the side was assuaged, the general condition of the patient was decidedly worse. She could not lie down, but was obliged to sit up constantly; the cough was exceedingly distressing, and the anxiety much aggravated; she occasionally rambled in her talk; the pulse was 100, soft, pappy, and intermitting, and the general strength very low. She was now ordered a table-spoonful of the following mixture every four hours.

℞. Extract. digitalis purpur. gr. iv.
Aquæ cerasi nigri...... unc. ij.
Plumbi acetatis........ gr. j.
Laudani liq. sed. ...... gtt. xv.
M.

Next (18th) morning, a decided amendment; the dyspnœa and cough less frequent; expectoration had commenced; some sleep during the night. The medicine to be continued, and veal broth to be allowed. From this time she gradually recovered her health.

*Case* 6. A thin debilitated man, who had suffered repeatedly from attacks of pulmonary catarrh, was labouring under severe harassing cough, attended with a copious muco-purulent expectoration, when he applied to Dr. R. The mixture, with digitalis, sugar of lead, and opium, was immediately ordered for him; and in the course of five or six days, he had nearly quite recovered.

*Case* 7. A woman, aged 32, after exposure to cold, was seized with violent pneumonic symptoms, to which were added repeated attacks of severe cardialgia. She was five months gone with child. She was bled largely, and treated with small doses of tartar emetic and nitre. The pectoral distress was somewhat alleviated, but that of the stomach much increased by the remedies; and, as the pregnancy was deemed a proper objection to very copious bleedings, the mixture, consisting of three grains of acetate of lead in six ozs. of infusion of digitalis, was ordered for her, in doses of a table-spoonful every four hours.

After the third dose, the dyspnœa was decidedly relieved—the pulse less frequent, but the cough still very troublesome. The cardialgia had not returned; the expectoration, which hitherto had been "cruentus croceus," was more of the character to which the epithet "coctus" is applied.

Although there was a relapse of the severe symptoms, which required a small venesection, and an increase of the doses of the mixture; and although abortion came on in a few days afterwards, the patient progressively improved; the expectoration had become easy and moderately copious, the pulse soft, and the general pyrexia much abated. A miliary, or apthous eruption had appeared on the hips, and in the mouth and fauces; but this also gradually vanished.

A febrile reaction threatened to set in on the following week; but its advance was checked by repeated doses of the liquor kali acet. in bitter almond emulsion, and by six grains of Dover's powder at bed-time. In the course of a short time she became, (to use the German expression) kernel-sound, sound to the very bone and marrow.

*Case* 8. T. P. aged 40, was seized on the 22d Nov. with alternate heats and chills, with severe pain in the side, increased by full inspiration, and with a strangling cough, which returned frequently in paroxysms of great violence; these paroxysms often lasting for a quarter of an hour at a time. The patient could lie only upon his back, and even in that posture was continually panting for breath. He was immediately bled from the arm, and a refrigerant nauseating mixture, with small doses of calomel, given frequently.

Under this treatment he went on improving, till the morning of the 24th, when he was found considerably worse; intolerable wandering pain, like the stabs of a knife through the chest,—cough harsh and very severe — skin parched, and occasional delirium. A blister was applied to the chest, and a mixture with camphor, opium, and nitre ordered to be given every second hour. Although some relief was obtained from these means, the pneumonic symptoms were not satisfactorily subdued, till recourse was had to the acetas plumbi, with opium.

About a dozen other cases, similar in most respects to those which we have detailed, are brought forward by our author to confirm the good opinion which he has formed of the effects of lead, opium, and digitalis combined, in inflammatory affections of the lungs.

Few English readers will be inclined to be of as great faith as their German brother; still we must fairly admit, that as the sugar of lead is undeniably known to possess very considerable sanative powers in hæmoptysis, it is but probable that in pneumonia, bronchitis, and hectic irritation, it may have a certain range of efficacy.

Our author informs us that he is disposed to believe that the remedy exerts its influence chiefly on the smaller and capillary vessels; and he therefore always premises bleeding and other depletory measures, in order that the morbidly increased action of the heart and larger arteries may be considerably reduced. He has derived very pleasing results from its administration in all cases where the quantity of sputa is very large; it seems to exert a direct astringent power on the mucous membrane of the bronchi.

In asthma, it has been also of great service, by relieving the distressing dyspnœa, and in facilitating the critical discharge from the lungs. A case of severe chronic cystitis is mentioned, where exceedingly good effects were obtained from its employment, after the ordinary treatment had utterly failed. Reasoning from analogy, he is led to anticipate the same advantages in sanguineous apoplexy, which is, in an especial manner, a disease of the arterial red blood capillaries. When there is a tendency to serous effusion, either in the brain, or into the substance of the lungs, the remedy is not to be employed; it is the "plastic," and not the "exhaling," action of the vessels, or to borrow the German phrase, it is the "hypercrystallizatio animalis" which is under the control of lead.

[The late Dr. Rush, of Philadelphia, highly praised the use of acetas plumbi in menorrhagia, threatened abortion, &c. &c.]—*Rust's Magazin für die gesammte Heilkunde.*

---

### II. Re-vaccination in the Prussian Armies.

About two years ago, a very alarming epidemic of genuine small-pox appeared in different parts of Germany, and threatened to commit great devastation both in civil and in military life. Several regiments lost a number of men, and it was observed, that the disease affected chiefly the young soldiers and recruits who were at and between the years of 18 and 24.

It was therefore an object of army-policy to investigate the history of this epidemic with all possible attention and accuracy, and to endeavour to devise some means for the extirpation, and subsequent prevention of this desolating scourge, which if not arrested might often paralyse the very sinews of war.

The military physicians recommended that a general re-vaccination of all the recruits should be forthwith instituted, whether the marks of a previous vaccination were found on their arms, or not. The government, ever attentive to the welfare, and efficiency of our armies, promptly acted upon the advice of the medical board. In 1831, when the small-pox epidemic broke out at Erfurt, two regiments of the 3d division were stationed there: 6020 in all were vaccinated at that time; and out of that number 2354, or more than a third, exhibited genuine cowpox vesicles.

In the 8th division 2784 were vaccinated, and of these, 925 proved to be quite susceptible to the virus.

During the following year, nearly a somewhat higher proportion was obtained; in one division, 1594 out of 3942, and in another, so many as 2535, out of 3234.

Now, all must agree, that those individuals, in whom the operation succeeded, were truly susceptible of the contagion of small-pox; the capability of receiving one poison being coincident with, and indicative of receiving the other; at least such is the prevailing opinion; and until it be contradicted by facts, it is a safe and feasible one. How great must therefore be the danger of such a fatal scourge as small-pox invading our armies, especially when regiments are crowded together in a narrow space, as is often the case during war!

Fortunately we have already proof that a preservative means, when properly and assiduously employed, exists in our own hands; at Erfurt it was found that not one of the re-vaccinated soldiers was seized with the contagion during the prevalence of the disease in that place.

We need no farther proof of the utility of the official order.—*Ibid.*

---

### III. Case of Sudden Death from Asphyxia during Intoxication.

A young beggar-woman went into a public-house to buy tobacco. There were two men sitting in the room, drinking brandy. They gave her a glassful, which she forthwith swallowed, and asked for more. She drank eleven in succession, and being now intoxicated she hobbled into the street and there fell down quite insensible. In this state, her neighbours carried her home, and laid her on the floor. She now vomited several times, but did not recover her consciousness; and about midnight, nine hours after the debauchery, she died. It appears that no regular medical assistance was procured for her; various means were used by the attendants, and among the rest, the liquor of pickled cabbages and the water of a "fosse d'aisance."* The authorities of the place ordered, that a diligent investigation of the case be immediately instituted. On examining the corpse, it was found to be that of a healthy young women, apparently about 23, or 24 years of age. The mouth was crammed full with lentils.

*Dissection.* In the larynx, just beneath the rima glottidis, ten or twelve lentils lay, and on opening the trachea

---

* To form an idea of the popular domestic medicine of this district of Germany [Merzig] we may state, that it is a common practice to collect the dried excrement of dogs, and steep it in brandy;—this is a sovereign elixir for diseases. Dr. Chevalier mentions the case of a woman who was suffering from hæmorrhagia uteri, and to whom her nurse recommended some of her own vaginal discharge, as an effectual restorative. O mores Germanici!!!

many more were found, mixed with a frothy discoloured fluid. The right bronchus, where it sinks into the substance of the lungs, was completely stopped up with them; in the left bronchus there were none at all. The right lung was of an unusually dark colour, and much black blood escaped from incisions into its substance—a few of the lentils had found their way into the small air tubes. The left lung was more healthy, although two or three lentils were found also in its substance. The heart and large vessels were normal in structure; but the blood was thinner than after a natural death, and its colour was rather blueish-black, or inky, than of the venous hue. The stomach was filled with lentil-pap, and there was a strong odour of brandy—the mucous coat was streaked with dark lines, and marbled here and there with red spots,—the intestines and other viscera were moderately healthy. The encephalon was then attentively examined; but no abnormal appearance was detected.

The questions for enquiry now were, whether the death of this poor woman was solely and directly attributable to the quantity of brandy given to her; whether the filthy remedies administered, had any effect; or lastly whether the cause of death must be sought elsewhere, viz. in the obstruction of the air passages from the accumulated lentils. If the last-mentioned cause be admitted to be the true one (and medical men must be unanimous on this score,) then we have to determine in what manner, had the lentils most probably been introduced into the larynx and trachea. To explain this, we have only to remember that the unfortunate woman had vomited or attempted to vomit repeatedly, during her excessive intoxication; part therefore of the egesta probably remained in the mouth and fauces, and upon the next violent inspiration, were sucked forcibly into the air tubes, which, as well as other organs had lost much of their irritability in the general coma: the horizontal position on her back, while it added to the difficulty of expelling the food from the mouth, favoured the resorption of it into the trachea. Such appears to us to be the legitimate conclusion, both from the testimony of the witnesses, and from the appearances found on dissection; that the immediate and direct cause of death in this case, was the plugging up of a considerable portion of the air passages, by the introduced lentils, and that the more remote and original cause was the stupefying action of the brandy, which not only induced the vomiting, but at the same time rendered this act dangerous, and in the present instance, fatal.

To determine the criminality of those who wilfully intoxicate others, belongs not to the physician, but to the judge. *Rust's Magazin.*

---

### IV. Experiments on the Medicinal Operation of Whey in some Chonic Diseases, and especially Phthisis Pulmonalis.

Dr. Kraemer of Munich, has communicated the results of his experience, during a period of nine years, in upwards of 500 cases of disease. He, like several other German physicians of late, has instituted an establishment for the reception of such patients, as may be considered to require the whey-regimen. The locality of this establishment is in a valley, partially surrounded by an Alpine wall; it is considerably elevated, being 2911 feet above the level of the sea; the air is cool, pure, and invigorating. The average temperature is not stated; but we are told that the heat during Summer is high, and that even in Winter, the cold is never very great. Dr. K. alludes to the importance of the freedom from dusty roads, and a loaded smoky atmosphere.

The almost instantaneous change in the feelings of the patient, who has left a crowded town for the unadulterated bracing air of the country, is frequently too obvious, to be gainsayed by any one. He breathes more lightly and easily; his night perspirations are diminished, and his appetite generally improves. True it is, that our hopes of such pleasing results depend on the nature and degree of the malady; for whenever there is a strong tendency to inflammatory attacks on the bronchi, or when the last, or colliquative stage of phthisis exists, the change of residence may be positively injurious, unless very special care and prudence be exercised. The same fears need not be entertained, when the lungs are sound, and the seat of the disease be in some other organ, as the liver, mesenteric glands, kidneys, &c. &c., even when the disease is accompanied with frequent congestive, sub-inflammatory relapses, or has already advanced to suppuration.

The whey that Dr. K. particularly recommends is that from goat's milk; it contains a large quantity of saccharine matter; nearly 5-8ths of all the solid contents yielded by evaporation—it has a fine flavour, which is derived from the aromatic pasturage of the adjacent mountains:—we thus procure a pure and grateful beverage from the hands, as it were, of Nature herself; impregnated with its own organic life, like the waters of a mineral spring; and free from the vile stuff of artificial composition! It appears however that the qualities of the goats' whey brought from different parts of a country, are by no means the same; the milk is much influenced by the character of the pasturage; and hence the importance of having an abundant supply in the neighbourhood of the convalescent asylum. The time at which the whey is to be drank is very early in the morning; the patient should begin with one glassful, or rather more than half a pint; and increase the quantity, to six, or eight glassfuls.

The good effects are speedily obvious; the cutaneous and urinary discharges become increased in quantity; the bowels act more freely and regularly; the juices of the body are cooler and more bland than before, and a certain degree of gently invigorating impulse is given to the whole system. The speedy action of the whey regimen on the secretions and excretions is only apparent, when the quantity taken is considerable, and at moderately short intervals;* when taken more sparingly, it seems to pass chiefly into the nutritive function. The quantity of perspiration is sometimes so greatly increased, that the patient is obliged to change his linen once, or twice during the day; the laxative effects of the whey vary exceedingly in different individuals, some being purged, and others scarcely affected by it; occasionally, but this is rare, it seems to have even a constipating effect; in such cases a regular action of the bowels must be solicited by the electuarium sennæ, the potass. supertartras, or by small doses of Epsom salts:—these mild remedies diminish the abdominal plethora and the congestive state of viscera, which so often give rise to costiveness.

Some physicians have recommended as an aperient, the whey prepared with tamarinds, or wine-vinegar; but Dr. Kraemer very justly states, that the beverage thus made much less palatable, and that thereby the patients cannot be induced to take a sufficient quantity for the requisite time; it is much better to attain our object by the use of mild laxatives, and give the whey in its simple unadulterated state. This is especially necessary to be attended to, in all consumptive cases, and in those connected with any congestive state of the abdominal viscera. With this precaution, Dr. K. assures us that whey is by far the most safe, as well as the most efficacious article of food, for removing all acrid inflammatory states of the blood, rendering it mild and sanative to the system, and yet affording a sufficient supply of nourishment for the support of the system; and a strong argument in its favour, is, that during its employment, we may have recourse to any remedial means, with the sure confidence that their effects will not be baffled by the dietetic regimen. How often do medical men neglect this important caution in the treatment of

* In leucorrhœa, chronic obstinate gleets, catarrh of the bladder, &c. its diuretic properties are usually well marked, and very beneficial.

chronic diseases, and especially of those of the pulmonary organs; while they are exhibiting all sorts of anodynes and antiphlogistic medicines, the patient is permitted to have a free unrestricted allowance of animal, and other heating food!! Can we hope to quiet the boiling wave, with a few drops of oil sprinkled on its surface, while all the time, the wind is heaving it into commotion? and yet the conduct of many physicians is as absurd. Perhaps they will tell us, that they have no expectation of the ultimate cure of the phthisical patient, and that therefore, it is scarcely necessary to deprive him of any thing, which he may desire; their reasoning is fallacious, and is certainly quite contradicted by our experience; for although we admit, that few, or no cases of genuine confirmed tubercula consumption admit of cure, it does not follow, that we cannot assuage the suffering, and slacken, if not arrest the progress of the disease.

Numerous cases have been brought to our institution, in which we at once declared the impossibility of a cure; and yet during their residence there, and the steady employment of the whey diet, every distressing symptom was relieved, except the cough and emaciation; the breathing had become easier, the night sweats had gone, the diarrhœa ceased, and the feverish irritability soothed, as by a charm.

This surely is a something of no trifling importance, which has been gained. We are therefore justified in asserting that even these incurable cases afford a strong proof of the admirable effects of the regimen, and afford a hope, which alas is a feeble one, that if ever a means of repairing the changes of the suffering organ be at any future time discovered, we have it in our power to assist and promote its operation, in a simple, safe, and efficacious manner. The following catalogue exhibits the chief diseases in which the whey diet has hitherto been found most useful. 1. All cases of hectic irritation. 2. Phthisical, or atrophial wasting; whatever be the organ morbidly affected. 3. Diseases of the heart, and of the head. 4. Obstinate chronic affections of the bowels and other abdominal viscera.* 5. Scrofula, rachitis, and all indurations of the glandular system. 6. Intractable cutaneous diseases and gout. 7. All nervous diseases, especially when attended with excitement. 8. Diseases of any of the uro-poietic organs.

It is scarcely necessary to state, that if the whey-regimen be useful in the above actually existing diseases, its sanative powers are infinitely more conspicuous against the predisposing and fore-running conditions of the system. How many a patient might be saved by a timely resort to its steady use! Dr. K. strongly recommends that, along with the prescribed diet, a tepid bath should be employed once or twice a week, and a light vegetable bitter taken daily. The formula for the preparation of his favourite tonic is thus:—take equal parts of the strained juices of the Veronica Beccabunga, Sysimbrium Nasturtium, Menyanthes Trifoliata, and of the Leontodon Taraxacum, and mix them together. The dose is from one to three ounces in the course of the twenty-four hours. The herbs are to be collected fresh, and a supply of the juices prepared daily. Such is the sum and substance of Dr. K.'s treatment. We must not however forget to remember the powerfully adjuvant influences of a quiet and cheerful residence, of a pure bracing Alpine air, and of regular clock-work habits.

He very candidly admits, that some of the patients who have already reached the second stage of consumption, are made worse by the change from their accustomed abodes to his institution, and very properly attributes the injury to the too keen and penetrating atmosphere of an elevated situation. The medical attendant must therefore exercise a prudent discrimination in the selection of the cases, which are suited to the change: nothing can be more injudicious than the practice which hitherto has been unfortunately prevalent, of sending the unfortunate sufferers abroad, when all hopes of saving them

* In cases of severe hæmorrhoids it is singularly beneficial.

in their own country had vanished. In concluding his remarks on phthisis pulmonalis, the author alludes to the occasional good which some patients derive from the inhalation of emollient and sedative vapours.—*Hufeland's Journal.*

---

### V. Enormous Steatomatous enlargement of the Uterus—Uterus weighing 21 Pounds.

A woman, aged 34, soon after her confinement with a first child, observed a swelling of the hypogastrium, attended with occasional pain, and with complete amenorrhœa.

The swelling continued to increase for six years, during which the catamenia were not seen; she supposed at first that she was pregnant; but she soon found it otherwise. Upon examining the abdomen, the tumour felt unequal and knotty on its surface, and it was painful when pressed with the finger; there was no symptom of tympanitis, or of ascites present; the os uteri was found per vaginam, to be round, and but little open; the body of the organ was indurated and weighty. Every four weeks the abdomen was puffed up more than at other times, and a mucous discharge escaped from the vagina. Her health became worse and worse, and she lingered out a miserable life of pain for eleven years. Shortly before her death a distinct fluctuation was felt under the skin of the hypogastrium; a trocar was pushed in, and a large quantity of bloody serum flowed out.

*Dissection.* Upon turning back the omentum, an enormously enlarged uterus came into view; when separated from its connexions, it weighed 21 lbs. [civil-weight.] The anterior surface of the organ was studded with upwards of twenty fatty growths, some small, but others as large as a man's fist. They were covered with the peritoneal coat of the uterus; when cut open they presented a beautifully smooth fatty appearance, like that of the finest tallow. The posterior surface was of a white colour; and when the substance was divided, it was found to be firm in texture, and about an inch thick in every part. The cavity of the uterus was filled with a brown-coloured substance, of a confused fibrous texture; its orifice was round and but little open, and a gelatinous mucus flowed from it; the ovaria were indurated; the other viscera were sufficiently normal in structure, although somewhat displaced by the enlarged womb.—*Graefe and Walther's Journal.*

---

### VI. Abnormal Flexure of the Colon.—Death.

A young lady, aged 19, had for several months suffered from repeated and very severe attacks of spasmodic pain in the bowels, accompanied with most obstinate costiveness. Her health gradually pined away, and at length she was confined constantly to her bed; the slightest motion, and even any sudden noise, caused burning and stabbing pains in the epigastrium, which sometimes lasted for several days at a time; the abdomen was neither hard, nor was it painful on pressure, unless this was forcible.

The alvine evacuations took place only every tenth or twelfth day, and then dreadful torments were always induced; no purgative remedy was of any avail. Her medical attendant subjected her to a course of animal magnetism for four months; her sufferings, we are told, were soothed, but her "flying life was not arrested"!!

*Dissection.* Upon opening the abdomen, no viscus was seen but the great gut; it was greatly enlarged in size, and when traced, it exhibited three distinct curvatures in the following way; the right, or beginning portion of the colon, having first mounted upwards, dipped down into the pelvis behind the uterus; it then took a bend, and rose towards the left lumbar region, and again descended, as the colon descends, to terminate in the rectum. A quantity of hardened scybalous fæces was found in different parts of the bowel, and also in the rectum, which though not quite normal, was not much affected. The other viscera were tolerably healthy.

No probable cause could be assigned for this curious malformation of the colon.—*Ibid.*

---

VII. Upon a curious Sort of Inflammation of the Outer Ear, occurring in Insane Patients.

This rather uncommon affection does not appear to have been noticed by authors, and yet six cases have lately come under the notice of Dr. Bird; they all occurred in men. The ear becomes hot, red, or blueish, shining, and painful: the swelling is variable—sometimes it is very rapid in its progress—at other times it is much more slow and tedious; the parts most affected are generally the cavitas innominata, scapha, and anti-helix; the helix is less so, while the concha, tragus, antitragus, and lobules are usually exempt.

The swelling continuing to increase, at length assumes the appearance, as if the half of an egg, sliced through longitudinally, was clapped upon the ear, and there stuck; in time it becomes so considerable, that it completely hides the concha, and even the incisura helicis. The dorsum auriculæ is but little affected; the ear projects forward, standing out from the side of the head, which adds to the unseemliness of the poor patient. From the commencement of the inflammatory process until the swelling loses its heat and painfulness, the period is three, four, or more weeks; its colour becomes now of a darker red, or of a purplish cast; the skin breaks in several fissures, and from these a yellowish serum exudes; after another week or so the tumour breaks at the top, and discharges a matter like black curdled blood; this discharge continues for a short time, and the swelling gradually goes down, exposing the tragus, antitragus, and concha, which hitherto had been quite concealed by the overhanging mass, but which had not partaken of its character;—the blue colour slowly disappears, and at length gives place to a more than usually natural whiteness; the affected part of the ear remains, in some cases, thickened, and somewhat hardened; occasionally it shrinks together, obliterating the cavitas innominata, scapha, and the anti-helix, and altogether disfiguring the organ. One or both ears simultaneously may be affected. With regard to the exciting cause of this curious affection, Dr. Bird is at a loss to account for it. He has never been able to trace it to any outward injury; and, indeed, the peculiar appearances which it presents are very different from those of an ordinary bruise.

In five out of the six patients, there were symptoms of active congestion within the head, the carotids beat violently, the scalp felt hot, and the meninges of the brain were probably inflamed; every symptom indicated great arterial fulness. It is probable, therefore, that the minute and delicate vessels of the outer ear may partake of the general plethora and increased action, and these would no doubt be increased by any outward injury, or even by frequently rubbing the organ, to allay the itching heat.

There is nothing peculiar in the treatment of such cases.—*Graefe's Journ.*

---

VIII. The Vapour-cave at Pyrmont, similar to the Grotto del Cane at Naples.

This cave is situated at a distance of about 800 feet from the well-known mineral spring of the same name. It is not a natural formation, but was hollowed out and fitted up, in the year 1720, by Dr. Seip, whose attention was drawn to the place by the quantity of suffocating vapour exhaled from the fissures of the rock. Similar streams of gas issue from many places in the immediate neighbourhood of the cave, and from the mineral waters themselves, giving them a sparkling and effervescing quality. The geological characters of the ground are, that it belongs to the variegated sandstone formation, and that it consists of marle and ferruginous sandstone. The stream of gas is constant, but the quantity issuing varies much at different times; so that the height of the gaseous layer above the surface may be from one to eight, or even twelve feet. It is generally highest soon after sunrise and sunset in clear warm weather, or at the approach of a storm—also during a hoar frost in Winter, during which the vapour atmosphere has stood so high over the mouth of the mineral springs, that children, and even adults, have fallen down asphyxiated while attempting to draw the water.

The gas is sour to the taste, and has a suffocative smell; the relative quantities of the carbonic acid and of common air vary, according to the level from the ground at which we collect the gas; at the bottom, it consists of 48 parts of pure carbonic acid, and of 52 of air—at three feet, the proportion of the former was only 36½; there is no admixture of any sulphureous gas. With regard to the effects which it produces upon animal life, we may state generally that it is a very exciting, momentarily-irritating, heating, and antiseptic agent; a pleasant tingling warmth is experienced in the limbs; if the person stoops down, he is soon sensible of the sourish taste, and of a pricking in his eyes and nose; he becomes oppressed and dizzy, his breathing is laborious, and the pulse is much quickened; these symptoms become gradually more and more aggravated, until complete asphyxia be induced.

The poisonous force of the Pyrmont Cave is, however, much inferior to that of the Grotto del Cane, or of the "Poison Valley" of Java, although the agent of destruction be the same in all, varying only in the relative proportion of its quantity. When the carbonic acid gas is nearly pure, or much exceeds that of the combined atmospheric air, it produces, if inspired, a spasm of the rima glottidis, and thus the entrance of the air is quite stopped up, and speedy death necessarily ensues. Such phenomena have been observed in animals taken into the Dog Grotto and the Poison Valley. The following experiments were made at the Pyrmont Cave.

*Exp.* 1. A rabbit was introduced. In 50 seconds it became convulsed; in a minute and a half it lay motionless, and as if dead. Being now taken into the fresh air it soon revived, and in the course of a minute or two had quite recovered itself.

*Exp.* 2. A two-years dog was next exposed. The breathing became laborious immediately; it tottered on its feet, and after a minute fell down, convulsed in its head and extremities—the lips, tongue, gums, and palate were purplish—the eyes open—the pupils dilated. The twitches of the legs became more and more feeble, the breathing shorter and more rapid, and soon altogether abdominal. After four minutes and a quarter, the dog was taken into the open air, and within two minutes had nearly recovered. This dog was afterwards again exposed to the vapour, and kept in it for 31 minutes; it was then taken out, and sprinkled with water and rubbed. In seven minutes it was again revived.

*Exp.* 3. A moderate-sized cat, after four minutes' immersion in the vapour, was taken out; it revived in two minutes. Being again exposed for 15 minutes, it was found to be quite dead.

*Exp.* 4. A six-years old shepherd's dog was then confined in the vapour. After being an hour and seven minutes there, the irritability of the wide-opened eye was altogether extinguished; but the breathing was still perceptible, by a feeble intermittent motion of the under jaw:—it became gradually weaker and weaker, but did not cease altogether until nearly three hours from the beginning of the experiment had elapsed.

It thus appears that the vapour of the Pyrmont Cave is only slowly fatal to animals of moderate bulk; the death induced by breathing carbonic acid seems to be not a painful one; a stupefaction speedily follows a short-lived excitement. Those who have willingly exposed themselves for a few minutes to its influence, state that they might have died in the most tranquil manner, had they not been soon removed from the narcotic atmosphere.

The comparative destructiveness of this cave and of the Grotto del Cane is well seen by the following table :—A dog was killed at the former in 2 hours and 52 minutes—at the latter in 2 minutes. A cat was killed at the former in $15\frac{1}{4}$ minutes—at the latter in $1\frac{3}{4}$ minute. A hen was killed at the former in $2\frac{3}{4}$ minutes—at the latter in $1\frac{1}{4}$ minute.

In consequence of the suggestion of Baron Graefe, a vapour-bath has been established, during the present year, at the Pyrmont Cave, and very considerable advantages are expected from its regulated use. When the vapour is still further diluted with atmospheric air, it may be employed with much service in phthisis, attended with mucopurulent sputa. Confined in contact with the skin, it is useful in removing paralytic weakness, and rheumatic or gouty pains; the perspiration is encouraged, and the cutis acts more healthfully. On a somewhat similar principle, sterility, amenorrhœa, leucorrhœa, &c. may be benefited by its use—also deafness, weak vision, obstinate coryza, and debility or cramps of any of the sensual organs.—*Graefe and Walther's Journal.*

---

### IX. Description of a new Lithotome.

[Litotomo, et Processo di Litotomia di dottore Francesco Gattei. Pesaro, 1832.]

The instrument consists of two parts, one of which is an ordinary staff, having a rather large solid handle of wood. In this handle, on its outer (*i.e.* corresponding with the convexity of the staff) side, there is a slit or groove, running somewhat obliquely from its extremity, down half its length; it inclines a little to the left side, and is deep above, becoming shallower as it descends. Where it ends, there is placed a screw, which is provided with a longitudinally-cleft or forked handle, to admit the point of the thumb to rest upon it.

The other part of the instrument is the cystotome, or gutting gorget. It is nearly as long, and somewhat of the same shape, as the staff; on its upper extremity there is a pin, which stands out from the back edge, so as to prevent the instrument from passing beyond the fork of the screw, and about three inches or so from its cutting extremity, there is a ring set upon the same edge, to admit the finger.

When it is used, the staff is to be introduced as usual into the bladder, and the external incisions to be made as in the lateral operation; the surgeon now feels for the groove of the instrument, and cuts upon it with the cystotome; when this is fairly admitted into the groove, he then introduces the other extremity of the cystotome between the two laminæ of the forked handle, and it is to be confined in this place by the thumb of the left hand, which grasps the handle of the staff. The fore-finger of the right hand is now to be inserted through the ring mentioned before; and while the handle of the staff, which hitherto had been resting upon the symphysis pubis, is brought down to the inner side of the thigh with the left hand, the cystotome is to be pushed on along the groove into the bladder, with the right one; the cutting blade cannot escape from the groove, as its upper or handle extremity is confined between the blades of the fork, and it is prevented from being plunged too deep, so as to perforate the bladder, or injure other parts within the pelvis, by the pin pitting against the forks, and by the cutting point of the cystotome being met by the knob at the end of the groove of the staff. If the section of the bladder is found, upon withdrawing the instruments, to be inefficient, it may be easily enlarged with a common scalpel; the operator is placed in the most favourable situation to adapt its extent to the special circumstances of the case. The great advantages of this lithotome are, that by means of it a clean and moderate-sized incision may be always made, with no injury either to the recrum or adjacent parts. Subsequent experience must determine its utility. No cases, wherein the operation was performed on the living body, are adduced by Dr. Gattei.

### Extracts from German Journals.

### X. CONTINUANCE OF LIFE IN A NEW-BORN INFANT, IN WHICH THE BRAIN HAD BEEN DESTROYED BY CRANIOTOMY.

The mother had been delivered by the operation of embryulsion, two years before; the conjugate diameter of the pelvis being only 2½ inches. The head having been perforated with the scissors, the two parietal bones were extracted, and the whole of the encephalon removed; the child was easily brought down and drawn out with the fingers. While the accoucheur was engaged with his patient, and awaiting the expulsion of the placenta, he was surprised to hear a whining noise proceed from the child, which had been wrapped up in a napkin, and laid aside in a corner of the room. At first he thought that it must be a mistake on his part, and paid no attention to it; but in two or three minutes the sound was repeated; and now upon opening the towel, he found that the mutilated child breathed feebly, and even moved its hands and feet; and once more gave out a whimpering cry. These phenomena were observed for a few minutes and then ceased altogether.—*Hufeland's Journal.*

[Let our readers consult the review of Dr. Kennedy's work in our present number.—ED.]

---

### XI. OCCASIONAL THICKNESS OF THE SAC IN FEMORAL HERNIA.

In a case lately operated upon by Dr. Angenstein, of Cologne, and reported in Rust's Magazine for January, 1833, the herniary sac was several lines in thickness, tough, and of a cartilaginous texture, and this character of the protruded peritoneum extended fairly within the crural aperture.

The surgeon, aware of the extreme rarity of such an occurrence, examined it most attentively, in order that he might be satisfied that it was the sac alone.

He deemed it proper to excise a considerable portion of it. The patient was a female, 52 years of age, of an exceedingly gouty constitution, and several arthritic tumours were scattered upon different parts of the body, behind the ears, on the sternum, &c.

Dr. A. attributes the thickened state of the body to an unusual tendency in the system to a deposition of matter. It is however right to mention that the hernia had existed 15 years, but had never been incarcerated.—*Rust's Mag.*

---

### XII. TREATMENT OF VENEREAL CONDYLOMATA.

As a matter of course, the removal of these very troublesome excrescences must be varied according to their character and situation. When they are pediculated, or even considerably projecting above the level of the surrounding parts, by far the most expeditious, and at the same time, a very safe method of treatment, is excision with the knife or scissors. If our patient be alarmed at all cutting instruments, the ligature affords a sufficiently convenient substitute. But not unfrequently they are too flat and expanded for the employment of either mode. In such cases Plenk's lotion is one of the very best applications we can use.

℞. Hydrarg. muriat. corros. ʒj.

Camphoræ, ʒss.

Alcoholis, ℥j.

It is to be applied with a camel-hair pencil, once or twice a day.—*Ibid.*

---

### XIII. FACIAL HEMIPLEGIA—EXTERNAL USE OF PHOSPHORUS.

The symptoms of this local palsy are well known; the mouth is drawn to the sound side, the eye is half-closed and weeping; the point of the nose is sometimes distorted, and the patient is often utterly incapable of moving the forehead, eyelids, and nostrils of the affected side; the motion of the eyeball, however, remains perfect; and the saliva usually flows more profusely than in health; but part of the food, especially if it be liquid, is apt to escape from one corner of the mouth. The temperature of the palsied parts is often lower than that of the other half of the face. The general health may be quite unimpaired. This hemiprosopoplegia may happen at any period of life; but in childhood it is very rare. The following treatment was successful in three cases.

℞. Phosphori, gr. vj.

Olei animalis æther, ʒiij. M.

The palsied parts are to be rubbed with this embrocation three or four times daily. After it has been used for a day or two, several places become sore, and then form scabs or crusts, which gradually dry and fall off. The rubbing must be renewed a second time when the skin recovers its soundness; and in severe cases the operation requires a third repetition. Generally after the first desiccation, the parts are found to have regained a slight power of motion, which increases more and more after the second and third rubbings. The use of the liniment causes very considerable pain, and a feeling of burning; but no evil effect has ever resulted from it.—*Hufeland's Journ.*

---

### XIV. Use of Iodine against Salivation.

Every medical man knows well how difficult, and yet how desirable a thing it is, to check a profuse salivation, whether it has been induced by mercury or not. Hufeland informs us that in iodine we possess the wished-for means. In seventeen cases it was employed with striking benefit; the severe smarting, the tumefaction of the glands about the mouth, and the profuse flow of spittle ceased after three or four days use of it; and the mercurial sores often healed up at the same time.

The dose usually given at first, was two grains in the course of the day; and it was increased to four grains, in the following formula.

℞. Iodini, gr. v., solve in

Spir. Vini, ʒij.

Aquæ Cinamomi, ℥ijss.

Syrupi. ℥ss., M.

Half a table spoonful to be taken every six hours—the dose to be gradually increased.

---

### XV. Poisoning from Eating the Capsules of the Hyosciamus Niger.

Two girls, each about five years of age, had been amusing themselves with the seed-vessels of this plant, which grew in abundance round their father's door, and no doubt had eaten some of them. Their parents soon observed a trembling of the limbs, a restlessness and confusedness in their speech and conduct, and a general oppression of the whole system. They were not aware of the true cause of these symptoms, and allowed the children to drink freely of milk. Dr. Burdach was called to them eight hours after they had eaten the pods. At that time he found them chattering incessantly, and without any meaning in their words; then they began to leap and dance, as in chorea, and all the time they seemed not to know any of their family. The hands, feet, and muscles of the face were every now and then twitched with convulsions; and so strongly did they struggle, that it was no easy task to restrain them, or to take away whatever they laid hold of; they bit and scratched and nipped every one who interfered with them. Sometimes they would grind their teeth, and push out their tongues, which were shaken to and fro with a trembling movement. If water was offered them, they drank freely and without any difficulty; but one of them always carried the jug upside down to her mouth. The eyes were sparkling, and constantly rolling about; the albugineæ were red, and the pupils so widely dilated, that the irides. seemed like a narrow circle; they were also insensible to light. The pulse was small, hurried and indistinct.

An emetic mixture, containing the tartrate of antimony, was immediately given, and repeated until free vomiting was induced; a quantity of hyosciamus seeds were among the egesta. A table-spoonful of wine vinegar was then given at intervals; and strong coffee allowed for drink. After this treatment had been continued for a short time, the symptoms began to abate, and the children recognised their parents. Milk and light farinaceous food were ordered. On the following day, they were both cheerful, and nearly quite well.

---

### XVI. ENCYSTED HYDROPS OVARII—SPONTANEOUS CURE.

*Case* 1.—A young woman, 20 years of age, who had been married for three years, but had never been pregnant, applied to Mr. Burdach, in consequence of a large tumour in the left iliac region. She stated that soon after her marriage, she experienced smarting pains in that part, and since then, that the swelling had gradually developed itself, without causing much inconvenience.

One day, having exerted herself much to lift a heavy weight, she suddenly felt, as if something snapped and gave way in her belly; immediately a watery discharge flowed from the vagina, the tumor sunk down; and there has been no sign of its re-appearance, now for 18 months since the event happened. The woman's general health is good; but she has never yet been in the family-way.

*Case* 2. A case in many respects very similar to the preceding one, is detailed in the able memoir by Dr. Montgomery reviewed in our present number. A woman separated from her husband, became affected with what was considered ovarian dropsy, and which enlarged the abdomen to the size of a six months' pregnancy; some of the other symptoms of this state were likewise present. After an attack of inflammation, during which it may be presumed, that the parietes of the tumor formed an adhesion with the upper part of the vagina, there took place suddenly a discharge of gelatinous fluid from that cavity, and the abdomen completely subsided in the course of a day; the previously entertained suspicion appeared to us to be confirmed beyond a doubt.

*Case* 3. The life of an innocent young woman was once nearly sacrificed by an occurrence analogous to the preceding two cases.

She had a large swollen belly, as if she was several months gone with child; but this enlargement suddenly gave way to a profuse discharge of fœtid matter from the vagina.

Unfortunately for her, there were two foundlings, who had died from exposure, discovered about the same time; suspicions fell upon this woman, and she was actually condemned as the infanticide. By the humanity however of several surgeons and physicians, who accurately examined the case, she was afterwards acquitted, and liberated.—*Cyclopædia of Pract. Med.*

---

### XVII. LIGATURE OF THE SUBCLAVIAN ARTERY BELOW THE CLAVICLE.

A young man received a sword-thrust through the folds of the axilla, in a duel. The hæmorrhage was checked by compression, and in eight days the wound was nearly healed; but now unfortunately the bleeding returned, and although restrained for the time, broke out a-fresh at different intervals. Professor Blasius of Hallé determined therefore to tie the subclavian artery, below the clavicle. The operation was performed on the 20th day after the accident; and although no particular difficulty was experienced in any of the steps, the patient had been so exhausted by the repeated losses of blood, that he died on the 2nd day after. On dissection, the axillary artery and vein were found uninjured; the source of the bleeding had been from the circumflexa humeri posterior, and circumflexa scapulæ, the wound having penetrated from behind, through the tendon of the latissimus dorsi, upwards and forwards. The subclavian artery, at the point of the ligature, was well secured.

Dr. B. very correctly condemns in severe terms the early treatment of this case. Why was the artery not laid bare at once, and a thread passed round it? No time should be lost upon such an occasion; the delay of even six, twelve, or eighteen hours may be most injurious; for if an inflammatory action, nay an inflammatory tendency be established around the wounded vessel, the risk of secondary hæmorrhage is tenfold increased. Dr. B. was called one evening to a young man, who had wounded his hand deeply in the morning; a bungling surgeon, who had seen the patient then, had crammed compresses and other trash into and upon the wound; a certain degree of inflammation had thereby already commenced, when Dr. B. applied the ligature; on the 4th day, the vessel had ulcerated; the bleeding returned; and a second operation was necessary. But should the wound heal partially at first, and the hæmorrhage not recur, till the 16th, 18th, or 20th after the accident, when suppuration had been established for some time, not only are the difficulties of securing the injured vessel greatly increased, but also, the chances of ulceration of its coats at the site of the ligature and consequent bleeding. The parts are much changed in their tissue, and are matted together, so that it is often not easy to distinguish between them; and moreover the artery is so glued to its sheath, &c. that it is scarcely possible to isolate it satisfactorily. Still, with all these disadvantages, the tying of the artery is much safer than the employment of any other styptic remedies; our prognosis however cannot be so favorable, as it would have been, after an earlier operation.—*Rust's Mag.*

---

### XVIII. Cæsarian Operation in Paris.

The woman was forty years of age; she had been eight times pregnant: the fifth and sixth pregnancies had terminated in abortions during the third month. In the remaining six she had been delivered by means either of the forceps, or of the crotchet; only two of the children had been born alive; one of them lived for eighteen months, and the other only for seventeen hours.

On the 3rd of February last she was again taken in labour. M. Bello visited her, and on examination of the abdomen, found that the uterine tumor was so prominent and depending, that the umbilicus touched the thighs, when the patient was sitting; the integuments were exceedingly stretched and of a purple colour, in consequence of the congested state of the superficial veins. Although she was still in the eighth month of pregnancy, the regularity of the pains indicated sufficiently the near approach of accouchement.

A consultation was held, and it was determined that delivery should be accelerated, before the patient's strength became much reduced. It was first attempted to replace and retain the uterine tumor in its proper place, that its contractions might bring down the child within the pelvis, to permit the application of the forceps; but the excruciating pains she felt in the region of the kidneys and in the ulcerated abdominal parietes (for they had cracked in several places) whenever any attempt was made to raise the tumor utterly prevented this plan from being followed. On examination per vaginam, it was found that the outlet was sufficiently large in all its dimensions; but the neck of the womb could not be reached with the finger, and this was not wonderful, when the os uteri was actually higher than its fundus; to effect the delivery by turning, was therefore out of the question. It was resolved that the Cæsarian operation should be performed; and this was done on the forenoon of the following day by M. Baudelocque. A living child was extracted; but it survived only seventeen hours. The patient bore the operation, which did not exceed sixteen minutes, remarkably well; while the dressings were applied she fainted away; but this did not seem to arise from the loss of blood, so much as from the agitated feelings of her mind. Fifteen hours afterwards she was a corpse.

*Dissection eight hours after death.*—The abdominal parietes were found remarkably thin and wasted; the fibres of the recti muscles were scarcely recognisable, the muscles having been quite atrophied and reduced to mere cellular tissue. The pelvic and part of the abdominal cavity were filled with clots of blood, which had stained the peritoneum of a purple colour. The cavity of the uterus also contained a good many clots; the incision had been made through its fundus and part of its posterior surface, for this surface had been the anterior one during life, and so completely had the womb been hanging out from the abdomen, that its anterior surface lay in front of the arch of the pubis. The urinary bladder was situated between the pubis and the vagina, which had been so much stretched, that the neck of the uterus covered the bladder.

The spinal column was quite normal, from the neck to the pelvis; but the angle formed by the lower lumbar vertebræ and the sacrum had become a right angle, so that, when the woman was in the sitting posture, she rested on the posterior surface of the sacrum, and not upon the sciatic tuberosities, for these were directed forwards and somewhat downwards. The effect of this must have been, that the trunk of the body was thrown much forwards and the shoulders backwards, and we can understand the reason of her constant position in bed, having the head and back supported with a number of pillows. The measurements of the pelvis were as follow;—the antero-posterior diameter, from the symphysis pubis to the base of the vertebral column, was four inches and eight lines*—the transverse was four inches and nine lines; the former was, therefore, eight lines longer, and the latter three lines shorter, than in a well-formed female pelvis. The oblique diameters were each four inches and four lines, or two lines shorter than usual. Of the outlet, the antero-posterior diameter, measured from the under surface of the symphysis pubis to the point of the sacrum, was four inches and two lines, and the transverse only two inches and nine lines, or an inch and a quarter shorter than it ought to have been.

The cause of the excessive protrusion of the gravid uterus was, no doubt, owing to the unusual prominence of the sacro-vertebral junction; and the repeated distention of the abdominal parietes, during so many former pregnancies, gradually so weakened them, that they could afford no support, but became extremely thin, and quite atrophied.

The reporter of the case, M. Bello, admits, that a child of eight months might have been brought through such a pelvis as this woman had, "per vias naturales," but that the existing circumstances at the time prevented the adoption of the plan. That the woman died of internal hæmorrhage is unquestionable; and, perhaps, the true reason of this accident was, that the incision was made through the fundus and back part of the uterus, where the placenta is usually attached.—[By the bye, how comes it that not a word has been mentioned about the expulsion or removal of the placenta? we are not told when or how it came away.] The same accident occurred to the late Mr. John Bell; but, in his case, the placenta was attached to the anterior surface of the uterus.—*Transactions Médicales.*

* It is afterwards stated that the vertebral column, joined on to the sacrum in the manner we have noticed above, was only two inches and a quarter distant from the symphysis pubis, and that the brim was divided into two unequal halves, of which the right was larger than the left.

---

### XIX. The Artificial Production of Cow-pox.

In 1831, Dr. Sonderland, of Bremen, endeavoured to prove the identity of the variolous and vaccine poisons, and to point out a method of producing artificially on cows the vaccine pustules. He took the bed-linen of a patient labouring under the suppurative stage of small-pox, and which, therefore, was well impregnated with the active virus, and enveloped the bodies of some young cows with them for twenty-four hours; they were laid on the backs of the animals, and made fast round their legs. When removed, they were then hung up before their mangers, so that they were forced to inhale the infected atmosphere. In a few days, the cows became feverish, and on the teats and other parts of body, where the skin is fine, numerous pustules, in every respect like to those of the natural cow-pox, and the matter of which, when inoculated, was found to communicate the disease as on ordinary occasions. If these statements be quite correct, the inferences from them are obvious, and exceedingly interesting; they would shew, that what have been hitherto considered two diseases, are essentially but two forms of one disease, modified by the different constitutions of man and of the cow; and they would explain why the cow-pox has of late years become less frequent in dairies, since the epidemics of small-pox have been in a great measure unknown, the chances of infection being less, equally to the beast and to mankind. Besides, an interesting field for observation and experiment would be opened up, to enquire whether any of the other contagious diseases can be communicated to the cow, or other tribes of domestic animals; and if so, what changes and modifications do they undergo?

M. Numann, director of the Military School at Utrecht, availed himself lately of an epizootic, which prevailed in the environs of that city, to repeat some of M. Sonderland's experiments—with what results we shall now explain.

A cow, four years old, was enveloped with the sheets which had covered a variolous patient from the beginning of the disease until the fourteenth day, when the suppurative stage was fully established. No change was found to have taken place till the sixth day, when he discovered three or four slightly elevated spots on one side—they were about the size of pepper-corns. On the eighth, ninth, and tenth days, a few more fresh papulæ were observed upon the back and haunches; they contained a little clear serosity on their summits, and this had all the appearances of ordinary vaccine virus. These vesicles gradually dried up, and were then covered with small crusts, which fell off in the course of a few days.

*Obs.* 2.—A young cow, two years old, was treated in a similar way. No particular change was noticed until the sixth day, when only one pustule could be discovered on the shaved portion of the back. On the following three days, two fresh papulæ appeared—they contained a little lymph, and then passed through the stages of desiccation and desquamation, as with the former cow. Probably, one reason that the subject of this experiment shewed a less perfect eruption was, that it was kept all the time in the open field, and not taken into the cowhouse.

*Obs.* 3.—A milch cow, six years old, was enveloped with the same sheets which had been used in the first experiment; this cow had, moreover, been vaccinated six or eight weeks previously with lymph, procured in the ordinary way. No febrile re-action, nor any appearance of papulæ or of vesicles, was observed; but whether the failure arose from the protection of the former vaccination, or whether the infected sheets, which had been used before, had lost too much of the virus, it would not be fair to decide from the result of a single experiment.

M. Numann was now anxious to ascertain, whether the lymph of the vesicles of the two first cows was capable of communicating genuine cow-pox to persons vaccinated with it. Three children, of eleven, seven, and four years of age were made the subjects of the experiment; the left arms were punctured in three places, and this new lymph inserted, and the same number of punctures were then made in the opposite arm, and ordinary vaccine matter introduced. On the third and fourth days following, the punctures on both arms were red, and nearly similar to each other. On the fifth, the punctures on the left arm of the eldest child were in the same condition; two only of the punctures on the left arms of the other two children were developed, the third puncture having been ineffectual.

On the 7th and 8th days, the irritation of the punctures on the left arms of all the children had passed away. The vaccination of the right arm was followed by two regular cow-pox vesicles in two of the children, and by one in the third; these vesicles underwent the usual stages.

The virus collected from the second cow was introduced by four punctures in the arm of a child two years old. For the first few days the wound became inflamed, and seemed to be advancing to suppuration; but, by the seventh, all these appearances had vanished.

M. Numann, from these experiments, is led to believe that it is not genuine vaccinia, which is caused by the infection of a cow with variolous matter;—he thinks that the sparing and very limited eruption which he observed on the backs of the cows ought to be considered rather as a modification, or an analogous sort of the genuine small-pox.

It is to be remarked that in M. N.'s trials, the animals suffered extremely little disturbance of health; whereas M. Sonderland has intimated the occasional severity of the constitutional symptoms.

[It is to be regretted that the author does not state distinctly whether the cows on which he performed his experiments had ever exhibited previously the eruption of the proper natural vaccinia;—it may possibly be, that their systems had obtained a certain degree of immunity, or protection in this way; at all events, the circumstance should be noticed in reports of such experiments.—*Ed.*]

M. Numann adds that it is a fact generally known that true vaccine virus oxidizes quickly some of the metals, and especially iron; a property not possessed by genuine variolous matter, nor by the lymph of the pseudo-variolous pimples on the cow; for one of the lancets which had been imbued with it did not present 14 days after the slightest trace of oxidation.

In conclusion, the interesting topic of this memoir deserves serious attention, and ought to be cautiously investigated in a much more elaborate style, and on a much more extensive scale, than has been attempted hitherto.—*Journal der Pract. Heilkunde.*

---

### XX. ON SANGUINEOUS TUMOURS OF THE CRANIUM.

The most common and least dangerous sort of these bloody swellings is, when the blood is effused between the aponeurosis of the occipito-frontalis muscle, and the common integuments.—They are very often observed on the heads of new-born infants, and are no doubt caused by the severe contusion of the cranium, during its expulsion through the pelvis. This is the "caput succedaneum" of some German authors. In general, it may be easily discussed under the use of resolvent applications.

The second variety of bloody tumors of the scalp, and which is usually caused by contusions or other external violence, is that which has been described by M. Zeller under the name of cephalæmatomié. The blood is diffused between the aponeurosis and pericranium. The German and Italian writers have often confounded this variety with the former;—it is only on this supposition, that we can account for their differences of opinion with respect to the danger or not of these bloody swellings, and to the treatment which they have recommended; some advising the knife to be used, others trusting to discutient lotions.

The fluctuation is not so distinct as in the first-mentioned kind, and the blood becomes diffused more readily, so that it does not generally present the appearance of a depression in the centre, and an elevated hardened border round; signs which have sometimes led surgeons to suppose that there was a depressed fracture of the bone, when the effects of the bruise were nothing but an ecchymosed subcutaneous swelling. In this sort the aponeurosis sometimes form a solid cyst round the extravasated blood. Whenever the pericranium becomes detached from the skull the injury assumes a more grave importance;—we cannot with certainty predict that the bone may not become ultimately necrosed. But this is rare, and authors have no doubt often committed the error of supposing that the blood was in contact with the bones, when the investing membrane of the latter was quite entire and firmly adhering.

M. Velpeau mentions a case of a child, only ten days old, being brought to him, for a supposed hernia of the brain.

A soft fluctuating tumor covered the greater part of the left parietal, part of the temporal, and almost the whole of the occipital bone. The dispersion of this swelling was easily effected in the course of a few days. It is quite an unusual occurrence, that the pericranium is detached from the bone in newborn infants, however difficult the delivery may have been, and however large the quantity of blood effused. Sometimes, indeed, when a true encephalocele does exist, we meet with bloody swellings, which have their seat next to the bone, on other parts of the head;—such cases are very generally fatal.

The third species of swelling is situated deeper than either of the preceding two. Chelius, in his Manual of Surgery, published in 1827 at Heidelberg, places it in the diploe of the bones; M. Velpeau thinks that it more frequently begins between the bone and the dura mater, although a case mentioned to him by M. Lanth is more favorable to the other opinion. A man received a blow with a cudgel on the parietal bone; but little notice was taken of it, and in the course of a few days he appeared to have quite recovered. Several months after severe pains were felt in the part diametrically opposite; (are we to understand the parietal bone of the other side?) and it was judged proper to trephine the bone there; but no correct information as to the true nature of the disease was obtained by the operation. After death, a fungoid mass was discovered, of the size of a large walnut, flattened, and, as it were, incysted in the diploe of the bone, where the blow had been received.

M. Velpeau has seen two cases in which blood was effused between the dura mater and bone during accouchement. It is very probable that the blood retained in this situation may undergo certain changes and ultimately give rise to some of the cranial fungoid tumors.—*Journal Hebdomadaire.*

---

### XXI. Use of Iodine in Apocryphal Swellings and Ulcers of the Throat.

Dr. Martini, of Lubeck, has found this remedy very successful in many cases of ulceration of the throat, in which it was quite doubtful, whether the venereal poison was the cause or not. A woman, 40 years of age, who had never, according to her own report, had syphilis under any form, consulted Dr. M. for an ulceration in the throat of two or three months' standing. The appearances of the sore were very suspicious; it was excavated, hard, and painful. The iodine, according to Coindet's formula, was ordered, and nothing else. In the course of a week the ulcer was much improved, and speedily afterwards it healed up. The woman's health, which of late had become very precarious, was much improved under the use of the medicine; and at the same time a leucorrhœa, which had existed for several years, ceased.

The author alludes to other three cases which occurred in male patients, all of whom had suffered previously from genuine syphilis. The subject of the first was a young sailor, who had taken a quantity of syrop de lafecteur, and syrop du cuisinier, (both of these nostrums contain corrosive sublimate) his throat exhibited several irregular excavations with elevated edges. The second case occurred in an old bawd; the greater part of the velum had already been eaten away; and the third in a merchant, 36 years of age, who had caught syphilis in England; was treated with mercury by one of the first physicians in London, and as was supposed, with success. He afterwards travelled in Russia and in France; the disease had repeatedly broken out under some secondary form; and a variety of treatments had been resorted to. To all of these three the iodine was given, and the results were most satisfactory; the ulcerations healed up quickly, and the general health of the patients was improved. In some cases the medicine requires to be persevered in for a considerable length of time, before its salutary effects are observed; but on no occasion has Dr. M. found any evil to result from its use.—*Journn. der Pract. Heilkunde.*

---

XXII. Use of Tartar Emetic in Croup.

*Case* 1. A child, 3½ years of age, was seized on the 1st of January, with the early symptoms of croup. On the 3d they had reached a formidable height; the shrill crowing noise during inspiration, the wide-expanded nostrils, the rapid heavings of the chest, the tossing and throwing back of the head to catch the least breath of air, in company with violent pyrexia, at once attested the disease. Eight leeehes to the throat were applied; a calomel powder given every hour or two, and a blister over the sternum.

5th. A similar treatment has been continued since last report; and under it the symptoms are much mitigated. Small doses of nitre, antimonial wine, and spiritus mindereri, to be given in the intervals between the calomel powders. Next day there was a relapse of all the alarming symptoms; the cough was frequent and strangling; the breathing laborious, shrill, and crowing, and the little patient was burned up with strong fever. Six leeches were ordered to the throat, and one of the following powders given every hour.

℞. Sulphuret. Antimon. rubri, gr. i.
Florum zinci, gr. ij.
Calomelanos, gr. vj.
Misce, et in pulv. vj. divide.

But the alarming symptoms were not abated, and they threatened a speedy death by suffocation, if relief was not promptly afforded. Two grains of emetic tartar, and ten of ipecacuan powder were divided into three doses, of which one was given every half hour till free vomiting was induced. Although this effect was not obtained, the breathing had become easier, and the little patient was not so agitated. Whenever the cough came on, much frothy mucus was expectorated—the pulse was also reduced in frequency, and with the exception of the great exhaustion, the case promised to go on favourably. Under the use of mild demulcent and expectorant medicines, the little patient recovered rapidly.

*Case* 2. Dr. L. was summoned to a child aged 4½ years, which had been labouring under the premonitory symptoms of croup for four days previously. The peculiar shrill piping sound of the inspiration, and the strangling cough, announced a case of the angina membranacea. Eight leeches were immediately applied to the front of the throat, and a powder consisting of calomel and Kermes mineral, given every hour. A blister was likewise put upon the neck. On the following day the child was found to be considerably relieved; but towards evening there was a relapse of all the very worst symptoms; and in consequence of the extreme exhaustion of the patient, a repetition of the bleeding was not deemed advisable; and the calomel, which had been pushed to the extent of twenty grains, had already been found ineffectual. The powders of emetic tartar and of ipecacuan, ordered in the former cases, were therefore given. No vomiting nor purging however were induced by them; but the distress in breathing became greatly diminished when two had been taken; the pulse was less rapid, and a gentle perspiration bedewed the skin. The emetic powders were continued, but now at longer intervals; and a small dose of calomel was given occasionally. On the following day the little patient was greatly better; the sleep had been quiet and refreshing during the night, the breathing not much hurried, and the cough less frequent and looser at the same time. From this date, convalescence might be said to have been established.

It is worthy of notice that in both of the preceding cases the tartar emetic and ipecacuan induced scarcely any vomiting; they acted as antiphlogistics and expectorants.

The third case mentioned by Dr. L. is one of ordinary bronchitis occurring in a child 20 months old; the treatment consisted in leeching, and small doses of the antimonial and ipecacuan powder; the cure was speedy and complete.

The fourth case is one of cynanche trachealis. Nothing except leeching and the exhibition of repeated doses of the emetic powder was done: but the success was most satisfactory.

In these two last cases the medicine caused much more vomiting and also purging than in the two others. Of late years the preparations of antimony, and especially the tartrate, have been highly recommended in pneumonic and bronchitic cases; to these diseases we may add all inflammatory affections of the other parts of the respiratory system.

They are admirably well suited to the treatment of young children, in whom we find difficulty of employing a multitude of remedies which may be used by adults. With a few dozen of leeches, and a phial of the ipecacuan and antimony powders, we may treat a host of diseases of the air passages, much more successfully than our neighbours, who are using remedies of every shape, consistence and colour, which the tricky art of the pharmacopolist can prepare.—*Ibid.*

---

### XXIII. Paralysis of the Nerves of Seeing, Hearing, and Smelling—Integrity of those of Taste and of Touch.

A young woman, 21 years of age, of a lymphatic temperament, was admitted on the 10th of March into the Hôtel Dieu.

As she was nearly quite deaf, and could not read, it was impossible to obtain any correct information of her malady. The physiognomy was motionless, the general attitude without animation, the eyes were fixed and prominent, and the speech was slow and difficult; she was constantly either complaining of a pain at the top of the head, or calling for food, or saying that she was in the family-way. Some symptoms of gastric irritation being present, a few leeches were applied to the epigastrium with relief. Her friends at this time informed the physician, that she had been afflicted with the headache for at least the six last years, accompanied with a gradual decay of hearing, and, of late, with a loss of the sense of smelling. The intellect did not seem to be directly impaired—the general sensibility of the skin of the face and head was entire—the voluntary muscles, also, of these parts were sound in their functions, but the sense of hearing was almost completely gone: the sight, which, upon admission into the hospital, was only much weakened, had since been lost, the pupils being dilated and motionless, and the conjunctiva, although evidently much inflamed and dry, from the cessation of the lacrymal discharge, scarce sensible to any mechanical irritation—the pituitary membrane, too, was robbed of its general and special sensibility. A probe, introduced into the nostrils, might be moved freely about, without any distress to the patient, and strong hartshorn did not, for the first few minutes, cause any sneezing; the sense of taste seemed to be unaffected. The intense cephalalgia increased daily in severity, the poor woman groaning continually and pressing her hands upon her head—at one time in a state of excitement, and the next moment in that of stupor or coma. After the lapse of another week, no decided change had appeared: the swelling and redness of the conjunctiva was, indeed, increased—the opacity of the corneæ greater at some points, and their texture more softened, near the junction with the sclerotic. On the 3d of May the patient miscarried, and died soon after from the profuse flooding.

*Dissection.* When the brain was taken out, the attention of the medical men was struck with the unusually large size of the cerebral nerves, which had been cut through. The meso-cephalon and the rachidian bulb were also much enlarged. The olfactory and optic nerves did not present any lesion in any part of their course. The pathetic nerves, the motor oculi of the left side, and the hypoglossal and glosso-pharyngeal, were quite sound. All the other encephalic nerves exhibited signs of disease; they were increased to at least three times their ordinary size, and numerous small spheroidal tumours, two or three lines in diameter, were developed in the interior of the nervous cords, or on their surface. Some of these masses were quite circumscribed, but not contained in any cyst; others were more irregular, and seemed to be formed of numerous minute granulations, deposited between the nervous filaments, which thus either were separated from each other or traversed the diseased substance. This was of a yellowish and opaque appearance, resembling what we see in partially-softened tuberculous matter. Most of the tumours were situated very near to the point of emergence of the nerves from the cerebral substance. These nerves, upon leaving the tuberculous mass, became suddenly diminished in size. The two motores oculorum proceeded from the summit of a conical mass resting on the pedunculi cerebri, whence the nerves arise. A similar appearance was found at the points of origin of the fifth cerebral nerves; the muscular portion of the right nerve appeared sound. On the left side, the tuberculous matter could be traced to the internal part of the Gasserian ganglion. A small tubercle was situated at the inferior part of the right sixth nerve, but the greater number of the filaments were above it, and did not seem affected. The seventh pair were diseased, from their origins to the internal auditory foramina. The right pneumogastric was in a similar state for the extent of an inch. The lungs of this patient did not exhibit any tuberculous deposits.

*Remarks.* If the description of the preceding case be altogether correct, some interesting physiological deductions might be gained. The opinion of Majendie as to the functions of the fifth pair is partly confirmed, and partly contradicted by the report. All the lesions of the organs of sight and smell, indicated by this great physiologist to be consequent on the injury of these nerves, existed in this case. M. Cruveilhier, indeed, who was present at the dissection, thought that he could perceive a small tubercle in one of the optic nerves; but the other examiners attributed the appearance in question to partial desiccation and exposure to the air of the cut surface, and, at all events, it was very indistinct. However that may be, we cannot refuse to admit, that the result of this case very beautifully corroborates the conclusion, that the fifth pair has a direct influence on the nutrition of the eye, and that, if it be not the immediate seat of the four special perceptive senses, it is at least intimately connected with the healthy development of their functions.

The most puzzling part of the symptomatology, is to account for the persistence of the sensibility and motility of the face, while the fifth and seventh pairs of nerves were so seriously involved; for, if any position respecting nervous physiology seems to be established by the phenomena of disease, it is that these two nerves preside over the above functions. Mr. Bell, who read a report upon the preceding case before the Anatomical Society of Paris, is of opinion, that the integrity of the functions was by no means so complete as is stated, for the very expression—"the physiognomy was motionless, the eyes fixed and projecting, and the attitude was inanimate," indicates that the energy of the seventh pair, which has been actually called, par excellence, the nerve of physiognomy, was much affected. Moreover, it is quite possible that the fibres of the nerve might be surrounded with a diseased deposit, and yet have remained but little disorganized. This explanation may also account for the persistence of the sense of taste. It is more difficult to understand how the functions of digestion and respiration were unaffected, while the origin of the pneumogastric nerve was diseased.

The following case deserves to be recorded in connexion with the preceding.

A man received a severe blow just beneath the left suborbital foramen. He was stunned at the time, and, on recovering himself, it was found that there was complete hemiplegia of that side of the face, extending from the crown of the head to the base of the lower jaw. The nostril had lost its general and olfactory sensibility; one lateral half of the tongue was paralysed; the sight however was intact, but towards the twelfth day after the accident an ophthalmia, accompanied with dulness of the cornea, and the formation of an albugineous speck on its centre, supervened. The eye had from the first lost its general sensibility, so that it might be pricked without any distress to the patient; and the secretion of the tears had ceased. The mobility of the left side of the face was unaffected, but mastication could not be performed on this side. His teeth, the patient said, had no strength, and the food distended the cheek, and required his fingers to push it to the other side. The hearing remained entire. From this report we observe, that nearly all the lesions which are caused by dividing the trigeminus nerve, were present in our case.—*Revue Medicale.*

---

### XXIV. On the Plica Polonica.

M. Brierre de Boismont, when he visited Warsaw, on the first invasion of the cholera, was anxious to collect some authentic intelligence respecting this singular endemic disease. But in the city itself it is by no means common; he therefore requested his friend, Dr. Macinkowski to communicate the results of his experience for several years past, and of these results we shall now give a short abstract. The gist of the whole may be thus shortly stated; that the plica is to be considered, rather as a symptom of, or attendant upon a vitiated state of the general system than as a local or idiopathic disease, sui generis.

We are informed that it is comparatively rare among the better classes of society, and that unfortunately a very general notion exists among the lower orders, who are notorious for their filthiness, that it is of no use to apply for medical relief to counteract this peculiar disease of their country.

Such is the true cause of the stubborn obstinacy of the greater number of cases; the constitution has been in fact long polluted, and the offspring of this taint, viz. the curious disease of the hair is rooted, like a poison herb upon a poisoned soil. Nosologists have greatly erred in classifying the plica among diseases of particular tissues; instead of investigating its relations with different morbid states of the system, they have begun by noting down its prominent existing symptoms and appearances, and have tried to deduce from these a theory to explain the various complications of the disease. The error has arisen partly from an old historical tradition, that the plica was imported into Poland in the thirteenth century by the Tartars: but here, as is too often the case with historical narrative, each succeeding author has copied his predecessor, without troubling himself to consult the earliest chroniclers of the event. During the dreadful incursions of these barbarians, they ravaged and desolated all around them; and upon leaving the countries of Poland and Russia, they dammed up many of the streams with the corpses of their victims, thus inundating the soil, and poisoning the atmosphere with pestilence. The effect of this was to occasion a formidable epidemic, but no mention is made of the plica at this time. M. Frank, following Sprengel, is therefore quite in error as to the origin of the disease; and we are therefore warranted by history in not believing that it was communicated contagiously to the Poles by their cruel invaders.

Indeed, the very doctrine of its contagion is quite contradicted by the observations of Dr. Macinkowski; not a single fact, he says, has ever occurred to his notice, to lead him to suppose that it is propagated by direct contact. Besides, it is much more consistent with its singularly circumscribed locality or habitation, to seek the true efficient cause in the influence of manners and social customs, of food, or of certain terrestial and atmospheric phenomena, which may be peculiar to the country, just in the same way as we account for cretinism among the Alps, and pellagra in the plains of Italy.

In reference to the first mentioned causes, it is interesting to find that a usage prevailed long before the thirteenth century, or even the introduction of Christianity into the country, which very evidently must have had some reference to this subject.

This usage, which was connected with religion, and known by the name of "postrzyzyny," imposed an obligation upon all parents not to cut the hair of their children before they were seven years of age, at which time the ceremony was performed and that of baptism together.

It is not very easy to give any rational explanation of this national custom: be this as it may, we not unfrequently observe in young children, at home, affected with scrofulous disease, a matting together of the hair; and until the scrofulous tendency of the system becomes less and less, the hair does not obtain a healthy development. This systemic change is not unfrequent about the period of life at which the ceremony of tonsure was performed by the ancient Poles. Some authors have stated that the plica was first known in Poland about the close of the sixteenth century, and have enumerated among its secondary symptoms a number of those which belong almost exclusively to syphilis; but as this last-named scourge also was introduced into the country at the above date, it is very probable that the two diseases might exist simultaneously, and often were not accurately discriminated from each other.

Leaving however this topic, we shall proceed to give a short description of the genuine plica; and although we set out with contradicting the common opinion of it being a "morbus sui generis, et loci," we deem it more convenient to allude to its outward and more visible signs on the hair and nails, before treating of the systemic disease, with which we believe it to be in all cases connected. The opinion, which was long very prevalent among medical men, that uncleanliness was the common cause of plica, is now abandoned; the single fact that soldiers are sometimes affected with it, would disprove it; for we all know the rigorous discipline maintained over them in respect to washing and so forth. True indeed it is, that neglect of personal cleanliness may cause a matting of the hair, but this is not true plica; it is the "fausse plique," and only requires proper attention to remedy it. In the former the hairs lose their natural and healthy qualities; they are no longer elastic, or shining; they acquire a marbled appearance, and although glued and netted together with a glutinous matter are not less dry than before, with the exceptions of the roots, which are soaked with the diseased secretion.

Such is a concise description of the true plica; the bizarre accounts of the hair becoming painfully sensitive, and bleeding when cut, are drawn from the author's fancies, not from the bed-sides of patients. The second stage of the disease commences when the diseased secretion ceases and healthy hairs spring forth, so as to carry forward and detach the matted web from the scalp;—when the young hairs are sufficiently long, we may readily cut away the mass of disease with perfect safety. A hardening of the nails is an occasional concomitant of the affection of the hair. In respect to the constitutional symptoms, we do not hesitate to assert that every patient is either actually at the time affected with some acute or chronic disease, such as exist in other parts of Europe, or exhibits signs of having recently suffered. Whenever the general health begins to mend, the local disease becomes less; the morbid secretion ceases, and the old diseased mesh is pushed forward by the sprouting of the new healthy hairs beneath. We do not sincerely credit the assertions of authors, that they have seen cases of genuine plica, in individuals who were otherwise quite sound; and our opinion is confirmed by the very admissions of by far the best inquirers, that the disease, before assuming its pathognomic character, always appears under the mask of some other affection.

The local disorder of the hair stands in the same relation to plica as the profuse perspirations do to the sweating sickness, the diarrhœa to cholera, or the dry parchment skin to pellagra; —these are all mere symptoms; but they do not constitute by themselves the diseases in question.

Perhaps we should be more correct in reference to plica, were we to regard the local affection as a critical event, just in the same manner as we frequently do many hæmorrhages, or profuse sweating, purging, depositions in the urine, salivation, &c. Certain it is, that we have seen towards the close of serious acute diseases an entanglement of the hairs supervene within the short space of twelve hours, and from that moment an immediate amelioration of the case, which before seemed hopeless, take place. Such examples have generally been met with in young healthy men, who were labouring under violent inflammation of the head or chest, or under those gastro-enteritic affections which precede and accompany severe fevers.

But it is more commonly during and after the existence of chronic diseases that we observe the eruption of plica to act as a wholesome derivative or critical discharge. A pedlar Jew had for several years laboured under derangement, and the nutritive functions had also suffered;—on a sudden the symptoms of plica appeared, and the reason and general health were forthwith restored. Dr. Malcz, one of the best physicians in Warsaw, mentioned the case of an officer's lady, which is instructive. She was considered to be phthisical by all who had visited her, and had become extremely emaciated.

No sooner did the hair begin to be diseased than Dr. M. recognized a decided amelioration of all the pectoral symptoms; when the plica was completely developed, her health rapidly improved. At the end of twelve months the capillary disease ceased, so that the entangled mass could be removed; and fortunately there was no return of the chest complaint. It has been stated by many authors that the plica is not now so frequent a disease in Poland as it used to be. No doubt this is correct, in regard to the large towns and among the military; and we attribute the decrease solely to the improved state of medicine, which has introduced a more vigorous treatment of diseases in general. Unfortunately however the hut of the peasant and the hovel of the artisan are still rife with this endemic. By far the greater number of cases arise as we have described above. In the most aggravated form, which is not often seen, the diseased secretion is astonishingly active, the hairs not only grow quickly, but they become enlarged in size, so as to resemble thick horse-hairs. The Museum of Anatomy in Warsaw contains some specimens truly remarkable from their length and thickness.

In such cases the secretion may be compared in its effects with the colliquative discharges of other maladies. With respect to the external exciting causes of plica, they may be endemic, or special and individual. It is a fact of history that the disease was at one time known not only in the adjoining countries of Hungary and Germany, but also in Alsace, in the Rhenish provinces, and in Belgium.

The excessive filthiness of the poor population, especially among the Jews, no doubt, favors its development, although we do not consider this, per se, to be capable of inducing it. The evil is made worse by the ignorant prejudices which lead them rather to encourage than to stop it, when any symptom of its approach appears; they suppose that it is not possible to prevent its course, and that no medical assistance can be of any avail; they therefore wrap their heads up warm, employ fumigations to them, and moisten them with irritating washes.

We have not sufficiently accurate or extensive observations to enable us to explain the probable aerial and terrestrial agencies. It is a subject well worthy of a diligent inquiry; for its satisfactory solution would contribute powerfully to facilitate the treatment of the disease, and perhaps ultimately to eradicate it completely. It is unnecessary to enlarge upon the different sorts of remedies which have been proposed; the grand indication in all cases is to find out the systemic derangement, and to endeavour to cure that; the local malady is only of secondary importance.—*Archives Generales.*

---

XXV. JAHRES BERICHT UBER DAS CHIRURGISCH-AUGENARTZLICHE INSTITUT DER UNIVERSITAT ZU BERLIN. Von C. F. GRAEFE. Quarto, pp. 39.

ANNUAL REPORT OF THE SURGICAL AND OPTHALMOLOGICAL HOSPITAL AT BERLIN.

By far the larger portion of this report, published under the direction of the celebrated surgeon of Berlin, is occupied with a description of his new ligature-instrument. We are not aware that either it, or any similar one has been much used in this country. The canula for polypus of the womb invented by the late Dr. John Clarke of London, approaches somewhat to that now proposed by Graefe, but as far as we know, its application has been limited to the tying of uterine tumor. We shall therefore attempt to give our readers an accurate description of the latter.

The instrument consists essentially of two pieces, which Graefe distinguishes by the names of the ligature-"carrier" and of the ligature-"tyer;" but for shortness sake we shall call them the "director," and the "serre-næud." The directors are made of different lengths, according to the depth of the part where the ligature is to be applied; there should therefore be a variety of them, from two to eight or ten inches long; and all so constructed, that they fit accurately the other part of the instrument;—they should be of the thickness of a small silver catheter. Along one side is a deep groove, extending from about a line or two from the point of the rod terminating there in a short canal, through the remaining portion, to within an inch of the handle-end, where it ceases altogether. This ungrooved part is bored in its centre to admit the screw of the serre-næud, and to the side of this central bore is a crescentic slit, which extends as deep as the female screw, and in which moves a narrow flat rod or plate, affixed to the handle of the serre-næud. The serre-næud therefore consists of two parts; one of which is the screw about an inch long, which is received into the female screw, or bore in the handle-end of the director; and the other, the forked appendage, which is composed of the marrow rod, [rather more than an inch in length, and received into the crescentic slit in the director;] of the proper fork, of a semilunar shape, to which the ligature is made fast, or, in nautical language, is belayed; and of a ring which embraces the screw, and allows it to turn round. These three parts are soldered together, so as to form but one; and as the screw passes through and is confined in the ring, the tout ensemble constitutes the serre-næud portion of the instrument. Now the mode of using this instrument of Baron Graefe is very simple;—the noose of a ligature is passed through the short canal of the director, and the two ends are brought up along the groove, and then made fast at the forked part of the serre-næud, the screw having been previously introduced into the female screw in the director as far as it will go. By unscrewing, we raise the screw, and along with it the fastened ends of the ligature; the noose thereby hanging out from the point of the director is pulled tighter and tighter; and thus we relax or tighten it as we choose, by simply turning the screw one way or the other; and as the length of this is an inch, the size of the noose is consequently reducible by two inches, a degree of compression much more than is required ordinarily; and if in any cases we wish for a still greater, all that is to be done is only to unfasten the ends of the ligature from the fork, draw them up a little, and at the same time turn down the screw to the bottom of its sheath; we thus command a power of compression to the extent of other two inches.

Every surgeon knows what difficulty there is often experienced in applying the noose of the ligature round the neck of a deep-seated polypus, as when it is situated far within the nostrils or in the womb. Dessault contrived an instrument to convey the ligature sufficiently deep, that it may be carried up to the pedicle or narrowest part, and with a slight modification Graefe approves of it highly. It is difficult to give an intelligible description of it in writing, unassisted with drawings;—we may only state, that two wires, bent at their extremities into small semilunar forceps-like curves, are confined close together by a tube or canula, through which they are passed. The ligature is inserted into the ring thus formed by the meeting of the two half-circles of the wires. On pulling back the tube, the wires by their elasticity start from each other, and thus permit the thread to escape. A very excellent representation will be found in the engraving which the Baron has affixed to his memoir.

This eminent surgeon has for several years past used the above described instruments, in a multitude of diseases; and he assures us, that by their means he has been enabled to apply a ligature with much more ease and efficacy, than with any contrivances which were before in use. They are exceedingly well fitted, for trying all sorts of polypi, whether situated externally, or in any of the mucous passages, as in the nose, ear, throat, womb and rectum; for applying ligatures round the large arteries, as those of the thigh and arm, and also the carotid and innominata; likewise round the spermatic cord in castration, and round the neck of umbilical herniæ of infants. He has found it useful in the radical cure of old fistulæ, such as of the anus; a strong ligature, or wire is passed through the inner one into the rectum and then drawn out, and applied to his apparatus; by the necessary compression, it slowly cuts its way by ulceration, through the walls of the fistula; an adhesive inflammation is meanwhile established, and a complete cure often effected, with little inconvenience or distress.

[We believe that this is the principle of the treatment which has been so long, and in some cases so unsuccessfully employed by M. Van Butchell of London.] For the removal of many cutaneous growths, the ligature is also admirably fitted; when they are at all pediculated, the manœuvre is simple; and even when the basis is broad, a few incisions round it, will enable the operator to effect his purpose easily.

Graefe has in this manner frequently removed large lipomatous growths, also nævi materni, and anastomosing aneurisms with perfect safety, when extirpation with the knife would have been dangerous. For these and many other cases, our author's instrument appears to us to hold out many advantages which entitle it to a preference over the different contrivances, that have hitherto been in common use. But we do not think it at all likely that English surgeons will ever be inclined to use any instrument, as a "presse-artere," but the simple thread, secured by a knot, and either cut close, or permitted to hang from the wound. Where it may be wished to extract a ligature, which has been a sufficient length of time round an artery, or any other part, and which by its presence in a wound keeps up irritation and prevents its closure, the apparatus of Baron Graefe, slightly modified, is exceedingly convenient. In the place of the screw part of the instrument, described above, a small windlass is fitted on to the female screw of the director; the ligature applied along the groove as usual, is made fast to the axle of the windlass, and as this is turned round with a neat handle, the thread is generally tightened. All therefore that requires to be done, is to tie the ligature hanging out of the wound to the windlass, and by gentle traction, it will often come away.

We select a case, or two, in which the instrument was used with success. A child eight months old, had a congenital nævus on the fore part of the neck, which measured two inches across, and projected considerably above the level of the surrounding skin. The risk of serious hæmorrhage, and the vicinity of the tumor to the air passages forbad the use of the knife, or of the actual cautery. The ligature was therefore the only safe means, which could be employed, to arrest the increasing size of the tumor, and even to remove it altogether; two, or three incisions were first made through the skin, round the base, and the ligature introduced fairly to the bottom of these, in order that a secure hold might be kept; as it was tightened once or twice every day, but never so much, as to cause much pain, or any threatening of convulsions; the sphacelated tumor dropped off in eight days, and within the fourth week after the operation, the cure was completed.

In extirpation of the testicle, Graefe recommends that the gland and cord be freely exposed, a ligature passed round the latter, and drawn by means of his instrument so tight, as to prevent the circulation through the spermatic arteries, and to benumb the sensibility of the nerves; the cord is then to be divided at about the distance of one third of an inch from the ligature; and the testicle may be thus extirpated without any risk of hæmorrhage, and with very little pain to the patient; should severe pains be felt in the seat of the ligature, some hours after the operation, we are not to remove it, but draw it as tight as we possibly can, so that a complete paralysis of the nerves may be induced.

Some surgeons have indeed condemned such a practice, and have stated as their chief objection the occasion which it not unfrequently gives to tetanus and other nervous affections; in the long experience however, of Baron Graefe, these dangerous symptoms have rarely supervened: and on the whole much less seldom, when all the cord has been inclosed within the ligature, as recommended above, than when it has been divided, and a long and often difficult searching after, and tying of the numerous bleeding vessels has been practised. He therefore gives a decided preference to the use of his apparatus which effects a complete compression, so that no hæmorrhage need be feared, and which being so small creates little irritation in the wound, and is easily secured in its place, by a strip or two of plaster. A case is mentioned where a polypus of the posterior nares, measuring three inches in length, two in breadth, and seven and a half in circumference, was successfully removed. A wholesome caution is given, in the extraction of these growths, which ought uniformly to be attended to, and the neglect of which has sometimes proved fatal most unexpectedly. When the polypus is so large, that it cannot be brought out by the anterior nares, the only alternative is to remove it by the mouth; now it has happened, that when it was detached from its pedicle, by the eating through of the ligature, it suddenly fell down into the fauces, upon the opening of the larynx, and thus caused death by suffocation; the Baron therefore advises that a spare ligature should be passed through the substance of the polypus, and its ends brought out by the mouth and fastened to the cheek in order that we may be able at once to extract it, when it has become detached.

If the polypus be situated very deep, we shall derive much assistance from using Dessault's, or Graefe's guiding staff, or canala, with a wire forceps point; indeed several of these may be requisite in operations on the womb, to enable us to carry up the ligature to different parts of the neck of the swelling; when we are satisfied that this is properly effected, they can be easily withdrawn, by merely screwing back the canula, when the continued wires, which previously had been retained together, start open, and thus loose hold of the ligature; the ligature is now to be drawn tight, by means of the apparatus, and the pressure increased every day, until the polypus is detached. The use of these guiding canulæ, is not sufficiently understood by English surgeons; they will be found greatly to facilitate the operation.

Appended to the description of his ligature-apparatus, are a few observations by Graefe, on the styptic qualities of a nostrum which of late years has acquired a high reputation among the Italian surgeons, and for the discovery of the secret of whose composition, not less than 300*l*. has been given, we are told, by Messrs. Godfrey and Cooke, Chemists, in London. Some experiments instituted at the Berlin Surgical Hospital in the year 1831, induced our author, to have a favourable opinion of its powers, but subsequent experience has convinced him that not much dependance can be placed in it, for stopping hæmorrhage from a large artery; unless it is uniform, or nearly so in its results, it would be dangerous to recommend surgeons to employ it, in lieu of a ligature, especially as we know that simple cold water will occasionally succeed, in cases where we should expect a frightful bleeding. It is supposed that this Binellian Water, (such is its name) contains a substance which the German writers call "Kresosot;" and in consequence of this, the Kresosot has been tried by itself in some experiments; but nothing satisfactory has yet been published. With respect to the French proposal of twisting the bleeding mouths of cut vessels, Graefe has made trial of it, in a few cases, but the results do not warrant him in approving of it, except where the vessels are small, and easily drawn out from their sheaths.

*Fractures of the Lower Jaw.*—Most of the cases of this injury have been treated without the application of any splints, or surgical bandages; the simple expedient of supporting the jaw, with a common handkerchief folded and applied under the chin, carried upwards, and tied on the top of the head, has been found quite sufficient.

*Fractures of the Ribs.*—The best bandage is a belt made elastic with twisted spiral springs, introduced between its folds: this yields gently to the action of breathing, and does not incommode the patient with any cordlike tightness. To prevent it slipping down, a shoulder-strap, or two should be fixed to it.

*Congenital Umbilical Hernia.*—Three cases have been treated in the hospital during the last year, and all with the ligature applied by means of the apparatus. They all recovered perfectly.

*Cleft Palate and Staphyloraphy.*—The operation was performed upon an adult patient two different times, but without success. He was, however, furnished with an artificial moveable velum palati, such as was recommended in the 12th volume of Graefe and Walther's Journal of Surgery; and this answered so well, that not only was his speech much improved, but he could swallow fluids with very little difficulty.

*Cæsarian Section.*—The unfortunate patient was 39 years of age, had suffered from rachitis in her youth, and her health had more lately been much reduced by frequent returns of menorrhagia. In 1831 she married, and soon afterwards became pregnant. The first seven months passed away without much inconvenience, but then, from time to time, she often experienced false pains, which were little attended to.

On the 2d of March, 1832, she was seized with regular labour. On the evening of this day she was first visited; it was then found that the first and second periods of delivery were already completed, and that the third period had commenced—the waters had also been discharged. The pains became more frequent and severe, but no progress was made. In this miserable state of suffering she continued for five days (shame to German midwifery!!); and, as there was no hope of the delivery being accomplished, the Cæsarian operation, as the only chance of saving the life of the patient, for no doubt had the child been dead some time, was proposed. All the surgeons, in consultation, agreed that embryotomy might possibly be attended with much difficulty (!!), in consequence of the narrowness of the outlet, and, under existing circumstances, that it would be still more dangerous than even the Cæsarian operation (!!) The operation was performed on the 6th of March, at noon. After the tegumentary incisions, the uterus was cut open in the direction of the linea alba, and the dead child was extracted by the feet. When the cord was divided, the placenta came away (by the vagina?) without any assistance, and the womb contracted firmly and sunk into the pelvis; a few stitches were then passed through the lips of the wound, and a bandage applied round the abdomen. On the evening of the third day, severe nervous fever, set in, and she died in the course of the same night.

*Dissection.* The greater part of the external wound had united; very few signs of inflammation were found in any of the viscera. The parenchyma of the fundus of the uterus was uneven, with many knotty indurations, and its outer surface was covered with large excrescences—there was no blood found within the cavity of the organ. The conjugate diameter of the inlet of the pelvis measured scarcely two inches, and the transverse four inches and a half.—[Why are the measurements of outlet not given? Our surprise, in future, at the frequency of this cruel operation in Germany cannot continue, when such a miserable and disgraceful state of professional ignorance is proved to exist even in, or in the neighbourhood of, Berlin.—ED.]

---

### XXVI. ON MUCOUS DISCHARGES FROM THE UTERUS.

[Traité des Maladies de l'Uterus. Par Mad. Boivin et M. Dugés.]

The second volume of the work of Madame Boivin and Monsieur Dugés contains a short chapter on the mucous discharges that issue from the uterus. Leucorrhœa may depend on discharge from the vagina alone, but the cavity of the womb not unfrequently contributes to its formation. When the speculum vaginæ is employed in the examination of females suffering from gonorrhœa, a glairy secretion in considerable quantity is often seen to flow from the uterine cavity. Of this we have satisfied others and ourselves. Our authors conclude that simple leucorrhœa most commonly originates in the uterus or in its neck. But observation shews that, in numerous instances, the vagina alone will furnish the secretion.

The mucous discharge from the uterus is always, according to our authors, the result of chronic inflammation. The division they adopt is that into sthenic or active, and asthenic or passive leucorrhœa.

*Subacute or Sthenic Leucorrhœa.*—This form of discharge is accompanied with a feeling of pain in the hypogastrium, extending to the groins, the sacrum, and the loins—a sensation of heat, and of pruritus at the commencement, and of smarting afterwards—scalding and pain in the discharge of urine—and sometimes febrile disturbance. The external organs sometimes exhibit the signs of irritation and of inflammation. On examination of the os uteri it is found perhaps more open, more soft, more moistened, and more painful than its natural condition would explain. The discharge at first is serous or sanguinolent, especially if it succeeds a bleeding from the uterus; it soon becomes thick and yellowish or greenish; sometimes it is glairy, sometimes more puriform and fluid. At a later period it is often milky and white, occasionally mixed with an almost transparent glair, or entirely consisting of the latter, which resembles the mucus secreted by the pituitary membrane. When this is the case, the inflammatory stage has passed away, a circumstance that happens, according to Blatin and to Pinel, in thirty-six or in forty days. Our authors have seen this termination happen earlier, but after the chronic stage has been established, the acute has again returned on the appearance of the menses, upon some excess, or even without any ostensible cause.

It is not always easy to distinguish this active form of leucorrhœa from the chronic or the passive. An experiment with remedies is frequently the best criterion, and discharges that would seem, from the absence of pain and of similar symptoms, to be independent of inflammatory action, are found to yield to antiphlogistic treatment. In the part of the country inhabited by M. Dugés, the females generally abstain from wine, and those who neglect this prudent precaution are subject to discharges, which a milder regimen and a beverage of water speedily disperse.

The treatment recommended by our authors is simple, obvious, and perhaps inefficient. It consists in hip-baths, lavements, emollient poultices, and fomentations, and the steady injection of a bland and tepid liquid, directed to the uterus by means of a tube from a reservoir, raised to a moderate height.

Such are the chief items of the methodus medendi which our authors recommend. The contributions of French practitioners are usually rather of a pathological than a remedial character. They frequently tell us the true nature of a malady, whilst we discover the manner of curing it.

A British surgeon or physician, if convinced of the existence of chronic inflammation of the womb, would probably venture on something more efficient than the lavement-practice of our Continental brethren. He would cup the patient on the loins—give small quantities of mercury, with conium, hyosciamus, or poppy—exhibit mild aperients, such as castor oil—and, as soon as pain had passed away, he would think of gentle tonics and sedative astringent injections. We have lately treated, with considerable satisfaction, some cases of discharge connected with, if not occasioned by, chronic inflammatory action of the uterus, in something like the fashion we have just described. One case made a strong impression on our minds. The patient, a young female, had suffered for several years from discharge, and had been under several practitioners without benefit. She suffered at times from pain in the loins, the digestive functions were imperfectly performed, and there was a tendency to slight pyrexia. Suspecting the existence of some affection of the os uteri, we examined its condition with the speculum vaginæ. We found the uterus somewhat anteverted, the os uteri thickened, the orifice larger than it should be, and the cervix presenting some tenderness on pressure. We directed small cuppings on the loins, a small quantity of the blue-pill, with extract of poppy and of rhubarb, occasional small doses of castor oil, farinaceous food, and the horizontal posture. Under these means, the pain, the pyrexia, and the functional disturbances were speedily removed, and only discharge with debility remained. For the former, we ordered an injection of the decoction of poppies, with lead—for the latter, small doses of infusion of calumba, with the carbonate of soda and the tincture of henbane. In the course of a month from the time we first saw this young lady, the discharge had disappeared, and the general health was restored. We mention this case in illustration of our treatment. Did our limits permit the interruption, we might readily adduce others of an equally favourable character.

We next advert to that which is described under the name of the chronic leucorrhœa.

*Chronic or Passive Leucorrhœa.* In many females, the discharge from the vagina has not had its rise in an inflammatory condition of the uterus, but depends, from the first, on relaxation and debility. The genital organs are habitually bedewed with mucus in such persons, and their linen is stained with a discharge, which they scarcely regard as a disease. Constitution and climate exert a great influence in producing this condition, which is said by our authors to be almost universal in moist and cold countries, as Holland, and some parts of Germany.

This form of leucorrhœa is much more unfrequent in youth than in later life. It is often the result of numerous confinements, or abuse of venery. It often co-exists, in girls, with chlorosis and amenorrhœa.

There are no symptoms of local irritation, unless no attention has been paid to cleanliness, when superficial excoriations of the skin result. The discharge is commonly lactescent. When the mucous discharge is abundant, it stiffens, as it dries, the linen that receives it, and stains it of a greyish colour deeper at the margin. Sometimes the discharge augments on the approach of the catamenia, sometimes it diminishes or ceases. Menstruation established in a case of amenorrhœa has frequently cured an old leucorrhœa, along with its attendant chlorosis. The discharge will vary in quantity, consistence, and in colour. When abundant it is commonly attended with some general and sympathetic symptoms, such as languor, emaciation, gastrodynia, and a sense of dragging in the loins and epigastrium. Sometimes there is sickness, sometimes there are pimples on the face and especially the forehead, but our authors seem to think that the severer symptoms are usually dependent on indulgence in pernicious habits.—Perhaps it may appear, that as passive leucorrhœa is dependent on debility, and as this is itself the result of many causes, the general or the other symptoms of disturbance that attend on the discharge must vary in different persons. As no fixed law can be given for the symptoms, so no decisive rule can be offered for the treatment.

Our authors advert to a curious and a difficult question. Will the leucorrhœa of the female produce an inflammatory discharge from the urethra of the male? They observe that this has occurred more than once, but they do not explicitly remark if it was observed by them. They think that the occurrence has been more frequent where the leucorrhœa was of the sthenic or subacute character. They also seem to think that in the reputed instances of gonorrhœa produced by the menstrual secretion, it was really occasioned by attendant leucorrhœa. Those most accustomed to investigate venereal diseases will feel the difficulty of deciding such a point. They will acknowledge how impossible it is to pronounce that a discharge is simply leucorrhœa.

Our authors devote but little space to the treatment of the malady. They place their principal reliance on tonics and astringents. The methodus medendi of this country is so much more ample than that of France, that we need not enumerate the remedies they mention. But perhaps we may observe that they recommend in an especial manner the black oxyde of iron in the dose of from three to six grains daily, taken before the principal meal. But all must be regulated by the circumstances of the case. Debility being itself but an effect, a rigid inquiry must ascertain its cause, and the combination of judgment and experience will be necessary to remove it. If the duty of analysis would permit us to allude to the results of our own practice, we would say that the injections of lead and of alum have appeared more beneficial than those of a stimulating kind. Attention has lately been drawn in this country to the powers of the injection of the nitrate of silver. We must say that although we have often employed it, we have seldom found it successful. The injection that has answered most generally and most completely has been the solution of the acetate of lead in decoction of poppies. Beginning with the strength of two grains to the ounce, we have augmented it to that of saturation, or at least to that of eighteen or twenty.

Two other remedies are deserving of attention:—cupping on the loins and the application of blisters in the same situation. Both have been productive of the happiest effects, but we think that the combination of the wet with the dry cupping adds to the efficiency of this mode of derivation.

Our authors relate three cases to which we shall briefly allude.

CASE 1. *Leucorrhœa of doubtful origin.*—Mad. de La * * *, æt. 40, had had several natural labours, and though delicate, was regular in the catamenial secretion, when she began to suffer from obstinate constipation and from leucorrhœa. Her sister had died of cancer of the womb, and this lady experienced, or thought that she experienced, symptoms analogous to those which her sister had exhibited. She had constipation, pain, and a sense of dragging in the loins, and a white discharge, at times abundant.

On examination with the finger, the cervix uteri was found of natural size and free from pain; it was turned to the right side of the pelvis and low down in it. With the speculum, reddish-brown spots on a nearly white ground were seen on the os tincæ. A thick and yellowish secretion issued from the orifice of the uterus.

A small caustic issue on each side of the sacrum, small doses of the sulphate of magnesia, and flannel next the skin, were the means advised by our authors. We are told that in a month the lady departed for the country greatly reassured as to her condition.

CASE 2. The second case related by our authors is one in which there was reason to suppose, that some serious disease of the uterus was going on. As this was indicated by obvious symptoms we need not pursue our allusion any further.

The third case is one in which there is more than reason to suspect that the discharge partook of a venereal character. It passed away under mercurial treatment.

We have noticed these remarks of our authors on Uterine Discharges, in order to excite the attention of our brethren to an accurate investigation of the causes of leucorrhœa. The treatment is frequently too empirical, and, influenced by a name, the practitioner not uncommonly fails to ascertain the real nature and origin of the malady. If he bears in mind its occasional dependence on different conditions of the uterus and vagina, and that these conditions are at times inflammatory and at times are not, he will probably adapt his means to the case, because he must feel convinced of the necessity of carefully studying the latter.

---

### XXVII. Confirmation of Sir Chas. Bell's Opinions on the Functions of the Anterior and Posterior Fasciculi of the Spinal Nerves.

Our attention has been recently drawn to a very valuable paper of Professor Muller, of Bonn, in a late number of the Annales des Sciences Naturelles. The experiments which he adduces are most satisfactory, and will be no doubt considered conclusive, even in Germany, where the doctrine of the separate functions of the abdominal and dorsal roots of the spinal nerves has not been altogether assented to. Meckel, Rudolphi, Weber, and others have admitted it, only as conjectural; our author himself performed some experiments in 1824, with the view of ascertaining its correctness, but the results were far from being uniform and decisive. Bellingeri, in Italy, was also engaged about that time in similar researches, and the conclusions which he drew were, that the anterior fasciculi presided over the sensibility, and the flexion movements of the trunk and extremities, while the posterior presided over the movements of extension. Even Mȧjendie, to whom the second seat of honor is due, as a physiologist of the nervous system, and along with him Desmoulins, in their Anatomie des Syst. Nerv. have not assigned totally exclusive functions to the two sets of nerves in question. Their own words are—"Si l'on galvanise l'une apres l'autre, une racine dorsale, et une racine abdominale, qui ne communique plus avec la moelle, on obtient à la verité des contractions par chaque racine. Mais les contractions par les racines anterieures sont en general plus fortes, et plus completes, que par les racines posterieures. Les racines posterieures pincées, tiraillées, piquées causent de la douleur, mais une douleur bien moindre, que cella qui resulte de l'irritation de la partie correspondante de la moelle. Alors aussi les muscles correspondans aux nerfs, dont on irrite une racine, se contractent; mais se contractions sont encore moindres que dans le cas de l'irritation meme de la moelle. La section d'un faisceau de racines dorsales cause une secousse de tout le membre correspondant. Les resultats sont inverses en operant sur les racines abdominales; leurs figures, leurs pincemens, produisent des contractions plus fortes et convulsives, tandis que les signes de douleur sont presque nuls. L'isolement des deux proprietés dans chacun des ordres de racines *n'est donc pas absolu.*" Muller commenced a new set of experiments on rabbits, in order to determine this most interesting question; but he found that the previous operation of opening the vertebral canal was so difficult, and attended with such excessive pain to the animals, as frequently to induce involuntary twitches of all the muscles even when the nerves were not directly irritated, so that he was precluded from deducing any satisfactory conclusions. Indeed there must always be this strong objection to all trials made on the higher animals; but the happy thought of Muller, to examine the spinal system of the frog, has fully compensated for the uncertainty of these. The vertebral canal of the frog may be opened with very little trouble, and with comparatively trifling pain ; the animal is so tenacious of life, that it remains quite lively after the operation, and the peculiar arrangement of the anterior and posterior fasciculi of nerves, further facilitates our investigations ; for these continue to be distinct from each other, and easily separable for a considerable distance from their points of origin ; the posterior root may therefore be raised on a needle and submitted to experiment, while the anterior one is free from all injury. We shall first mention the effects of simple mechanical, and then those of galvanic irritation on the two sets of nerves.

1. When the posterior root is divided the animal appears to experience 'quelque douleur ;' if the distal, or unattached portion be now seized and irritated, there is not the slightest trace of movement in any of the muscles of the trunk, or of the extremities. When the anterior, or abdominal root is simply touched, convulsive movements of the extremities immediately follow. The same phenomena, only more violent, are observed when this root is cut and irritated.

2. The galvanic experiments were performed at first with a single pair of zinc and copper plates. Upon applying the two plates to cut ends of the anterior roots, the muscles became convulsed ; but no such effect was ever produced when they were applied to the posterior roots. This latter position contradicts therefore the assertion of Majendie and Desmoulins ; but we must remember that their experiments were performed on mammiferous animals ; and in these the two sets of roots are too short to enable us to separate them satisfactorily from each other, and thus to avoid the irritation of one set, while we are experimenting upon the other. Even in the case of the frog, it is necessary, for the sake of accuracy, to isolate the one from the other by means of small glass plates ; because the galvanic irritation of the motor nerves is found to take place at the distance of half a line. But in order to insure perfect accuracy, it is better to employ a small voltaic pile ; for then we may either apply both poles to the cut end of the nerve, or we may apply one there, and the other to some of the muscles. The following are the results of Muller's experiments in this way.

1. When the two poles are applied to the posterior roots, no convulsive movements follow. 2. When one pole is applied to the posterior nerve, and the other to some muscle at a distance, slight movements of the muscles which are situated in the tract of the galvanic current are observed. 3. When the anterior root is made the subject of these experiments, convulsive movements immediately occur, whether both poles are applied to the nerve, or only one, the other being applied to a muscle ; and these movements take place not only in the muscles which are situated in the tract of the current, but throughout the whole extremity. 4. The same result, viz. the occurrence of convulsions, is obtained when one pole is applied to the posterior, and the other to the anterior root. We may therefore safely draw the conclusions, that the posterior roots of the spinal nerves never directly and of themselves provoke muscular contraction ; that when they seem to do so (as in the 2nd result) it is only from their acting as conductors, just in the same way as any other moist animal substance, of the galvanic current ; and lastly that the anterior nerves not only are conductors of the galvanic current, but also are excited thereby to induce muscular movements in the direction of their branches. Now one of these anterior nerves may be deprived of its "vis motoria," and yet retain its conducting power :—to exhibit this, we need only seize and compress it firmly at a little distance from the cut end ; and we shall find that no irritation, either mechanical or galvanic, applied between the point of compression and this end will induce any contractions ; but if one of the galvanic poles be applied to the end and another to a distant muscle to which the nerve is distributed, then contractions will immediately follow, just as if there was no intermediate pressure ; shewing thereby most distinctly that the nerve retains its conducting power.

It has been supposed that galvanism acts as a special and peculiar irritant to the nerves, and in a manner altogether different from mere mechanical injury; but this is not true, for any foreign body, even not metallic, such, for example, as a quill, when applied to a motor nerve, will provoke muscular contractions. Muller, from multiplied observation, has been led to conclude, 1, that galvanism acts upon the nerves like any other extraneous agent—2, that it is not the proximate cause of muscular contraction; but only that it irritates the nerves, and provokes their "vis motoria," which is altogether different from a galvanic power—3, that it has not been proved that nerves are better conductors of galvanism than other moist animal substances—4, that galvanism excites movements, only when a muscle or a motor nerve are situated in the tract of its current—5, that there are some nerves which have no moving power, and can never of themselves induce any movements; that these are only passive conductors of galvanism—6, that there are other nerves which induce muscular movements, not only on the application of galvanism, but also of any mechanical irritant—7, that the dorsal or posterior roots of the spinal nerves have no "vis motoria," but that the anterior have, and that, from these last, all the motor fibres of the conjoined spinal nerves are derived. He once more alludes to the fallacy of believing that the posterior are ever motor nerves, merely because, when one pole is applied to them, and the other to a muscle, certain movements take place.

The next object of his investigation, was to ascertain what effects are produced by irritation of the proximal, or attached ends of the divided anterior and posterior roots. He found that, when a mechanical agent, or when both poles of a galvanic apparatus, were applied to any of these, no muscular movements were ever induced; but that, when one pole was applied to the portions of the roots adhering to the extreme part (cauda equina?) of the spinal marrow, and the other to some anterior part of the body, as, for example, the head, the muscles of the trunk and extremities were thrown into convulsions. In one experiment, he divided all the anterior and posterior roots as high as the cervical portion of the marrow, and then gently lifted out the spinal cord from its canal, and laid it upon a small glass plate; upon applying both poles to its sacral extremity, there were movements in all the parts which had been left connected with the marrow, viz. the neck and anterior extremities. If this position be confirmed, it would shew, that the spinal cord is not to be considered as only the "ensemble" of the nerves which issue from it; for we have seen that the portions of the roots which may be left adhering to the extreme parts of the marrow, do not, upon any irritation, induce muscular movements, but that the marrow itself, if irritated, does.

A few cursory remarks on some of the cerebral nerves are appended to the preceding valuable memoir. Muller agrees with Mayo and others, that the portio dura is not solely and exclusively a motor nerve—when irritated, the animal seems to experience pain. The infra-orbital nerve is one of mere sensation—it has no "vis motoria." With regard to the nerves of the tongue, Muller is led, by his experiments, to state that the lingual, or ninth cerebral nerve, when irritated or galvanized, provokes violent convulsions of the member; that the gustatory, or third division of the trigeminus, excites none of these phenomena, either by mechanical or galvanic agency, except, indeed, when one pole is applied to the nerve and another to the tongue; but, as we have explained before, this sign is quite fallacious, the nerve serving only as a conductor. The glossopharyngeal nerve, on the application of both poles, excites convulsions in the pharynx. These experiments accord with those of Desmoulins and Majendie.

When the lingual nerve was cut, the animal (a cat) seemed to suffer pain; and hence Muller believes that this nerve, although chiefly a motor, is also, in some degree, a sensitive nerve, in the same manner as he supposes the portio dura and par vagum to be. But his experiments here appear to us far from satisfactory.

*Sir Charles Bell to Dr. Johnson.*

My dear Sir,

I am really obliged to you for giving me the perusal of the analysis of M. Muller's experiments on the nerves. I feel convinced that I was right, from the commencement, in building upon the anatomy, and not upon experiments. All that I ever valued, as ascertained by experiments upon living animals, was, that one nerve differed in function from another. As soon as this was determined, we had a key to the explanation of the nervous system, and had only to be diligent in the pursuit of the anatomy, and in the observation of the natural functions and of their disorders, when the roots of the nerves were affected with disease.

For see what the medical profession has submitted to? We have one man who, by experiment, finds that the optic nerve is not the nerve of vision, and gives to the fifth the function of all the senses:—Another makes experiments upon the brain, and finds that one part makes the animal go forward—that another part makes it go backward, and another makes it wheel round—incapable, at the same time, of comprehending that any one of these *actions* requires the union of all the functions of which they are in search. Then we have experimenters, who tell us that the anterior roots of the spinal nerves are for flexion of the body, whilst the posterior are for extension. There is another of our favourite physiologists,* who, by experiment too, has discovered that there is a fluid moving backwards and forwards along the spinal marrow and the ventricles of the brain, which if it chance to become cold, produces ague—by operating upon it, he can tame the most ferocious beast; and, to cure a horse affected with "immobilité," he applies a flaming moxa to the animal's back, to draw this fluid out from the ventricles of the brain! In a state of the profession where these things are received as matters of science, can any one diligently labour from the desire of earning its approbation?

When I had yesterday the pleasure of meeting you in consultation, I shewed you a preparation which I value more than all the contributions from experimenters. It is a dissection, by Mr. Newport, of the nerves of one of the articulata, in which the analogy of the great central cord with the spinal nerves of the vertebrata is made out. In one aspect of the cord, you see the nervous matter forming a succession of ganglions; on the other, you see a column of nervous substance running over these ganglions, in the whole length of the cord. Can there be any doubt that these different columns are for sensation and motion?

But I am omitting to express the pleasure that I have had in reading your digest of Muller's observations. He has with great acumen shown the misapplication of the galvanic influence—the mistakes that experimenters have run into by not distinguishing between the vital power of the nerve, and its power of conducting galvanic influence. With respect to the experiments on the nerves of the head, we have again the proof of the superior importance of anatomy. Is it not really provoking to find men announcing experiments upon the par vagum, the portio dura of the seventh, the ninth, the glosso-pharyngeal, not only without studying the functions of the parts to which these nerves go, but totally negligent of the associations formed at their roots, and that they are *compound* nerves? I have occupied myself, in the intervals of business, now for a long time, with the investigation of the anatomy of these nerves, to show from whence the sensibilities of the throat and tongue are derived—how the associated actions of respiration, speech, and deglutition are formed; but here come gentlemen, without troubling themselves with these matters, who, with the cut of a knife upon a living animal, seek to solve all the difficulties at once. Even in regard

* Majendie.

to the portio-dura, without noticing its connexions with the fifth pair within the bone, without noticing that a *sensitive* nerve runs into its very centre before the ear, they occupy themselves with experiments which are to declare whether that nerve be sensible or not; and they come to conclusions directly at variance with what we now happily know, through the daily observation in hospital practice, to be the distinct functions of the portio dura of the seventh and of the fifth. I stand excused if I harbour a suspicion that some of these gentlemen are not altogether candid. For many years before I ceased lecturing in Windmill Street, when treating on the nerves, there were hung up behind me, three large sheets or plans, one explanatory of the symmetrical system, or the fifth pair and spinal nerves, another of the more irregular and respiratory nerves, and the third of the sympathetic system. By first teaching the simplicity and order which belonged to these systems separately, and then placing the one sheet over the other, I conveyed to the pupils a lively conception of the intricacy of the whole, and yet how possible it was, by taking correct notions of the roots of the nerves, to bring order out of confusion.

In the plan which exposed the symmetrical nerves, it was made obvious that the head had the same supply of nerves as the body—that a nerve of the same structure with the spinal nerves, having the same double roots from the projected columns, having the same ganglion on one of the roots, performed the same offices with the spinal nerves: and that this nerve, in the system of Willis, was what is termed the fifth pair of the head. The experiments which were made to prove the correctness of the deductions from the anatomy, were exceedingly simple and conclusive. Your late friend, Mr. John Shaw, immediately after an ass was knocked down, irritated the roots of the fifth pair of nerves, when the jaws closed with a violent snap—the fifth pair was, therefore, proved to be a muscular nerve. Next, considering the sensibility of the lips, the infra-orbitary nerve was divided, after which the spur or the needle applied to the lip, produced no effect. The matter being thus settled, both from my experiments on the roots of the spinal nerves and from those on the fifth pair, which confirmed them, that the nerves of the symmetrical system were sensitive and muscular, it remained only to be determined what was the intention or use of the distribution of certain other nerves which I early in these enquiries called the "superadded" nerves to organs already supplied by the spinal nerves and fifth pair: and to this, the most difficult and important part of the subject, I applied myself. The conclusion to which I came from studying the anatomy, the functions of the organs, and the influence of morbid structure, was—that the seeming intricacy of the nerves resulted from there being somewhere a centre in the nervous system, which governed the actions of respiration; and that these nerves, diverging from that centre, passed on to the different parts which co-operated in the act of breathing. The first part of the subject, the symmetrical nerves, I conceived that I had put, by my lectures and publications, out of all hazard of contradiction, and had laid in it a solid foundation for further investigations; —the latter was full of difficulties, and yet so important to the explanation of a variety of phenomena of disease, that it seemed worthy of the united exertions of the profession. On part of this latter subject, as I have informed you, I am still employed, though with less advantage, since I have been obliged to attend to practice instead of giving lectures, where the successive demonstrations of the nervous system continually suggested new subjects of enquiry.

In due time, the facts which I had ascertained were announced in the Philosophical Transactions of the Royal Society, and ultimately in a volume compiled from the various papers in these Transactions. I do think that it is not creditable to the medical profession that the works professing to be systematic and to give a correct view of discovery, should have brought forward as authorities the names of mere experimenters—who first attempted to take the merit of these observations to themselves, and failing, have prosecuted the subject only to the effect of distracting the minds of those who have not perused any original papers nor the appendix of cases—who, in fact, are not aware of the labour and care with which the first principles have been laid down in them.

I am, my dear Sir,
Very truly your's,
CHARLES BELL.

30, *Brook Street*,

2*d Dec.* 1833.

---

### XXVIII. On some Malformations and Diseases of the Œsophagus.

It is a question, still subject to dispute, whether the fœtus is nourished by the umbilical vessels only, or whether the water of the amnios is at the same time swallowed, to afford a pabulum to the young being. The monstrosities of the œsophagus may, if properly studied, throw some light on this curious subject of embryology. The chief *"vitia conformationis"* hitherto observed are, 1. A complete absence of the tube. 2. An arrested development of some portion, generally the inferior. 3. A separation into two portions, an upper and a lower, by a horizontal septum. 4. Its division into two branches. 5. Its origin from the trachea. 6. Its separation into two distinct portions, which are united by a small firm cord. When the œsophagus is altogether wanting, the pharynx usually terminates in a cul de sac. When an arrest of development has taken place, the tube terminates in a cul de sac, lower down and more or less remotely from the stomach; and there is no cardiac orifice of this organ. Professor Rossi saw one case, in which the œsophagus was obstructed by a transverse septum, immediately above the cardia;—the child had voided meconium, before its death, which occurred on the third day after delivery. When the œsophagus is divided into two tubes, these may either be reunited into one, or may remain distinct, only united by a fibrous tissue. This latter case is extremely rare; and so is the strange anomaly of the œsophagus arising from the trachea. Dr. Mondiere has met with only two examples—the infants lived for about 20 hours.

The conclusions which may be fairly drawn from the study of these monstrosities are, that the swallowing of the liquor amnii is not at all requisite for the full development of the child; that the meconium is not at least the exclusive residue of the liquor; and that probably the upper portion of the œsophagus is formed before the lower, the development taking place, from above downwards; for no case has ever been known, where the lower portion existed, while the upper was wanting.

There is considerable analogy between the malformations of the œsophagus and those of the rectum.

#### *Rupture of the Œsophagus.*

It occasionally happens that the œsophagus becomes ruptured; but we may suppose that some previous ramollissement or gelatiniform change of its texture, has always pre-existed. A spontaneous rupture of a healthy œsophagus must be exceedingly rare. A case mentioned by Boerhaave seems however to deserve to be considered as such; the laceration was complete, there was no appearance of erosion or ulceration, the stomach was much distended; and during life there had been violent efforts of vomiting.

#### *Dilatation of the Œsophagus.*

This accident may be either congenital or acquired; it may affect the whole, or only one, or several parts of the tube. Vicq. D'Azyr found in the body of a man a distinct and well-formed crop, (as we see in birds,) without any other signs of diseased change; but in most cases the dilatation is owing either to a hernia of the mucous membrane through the muscular coat, or to a mechanical distention from a foreign body lodging in the passage, and, in short, to any cause which offers an impediment to swallowing. When the food is arrested in some part of the gullet, it undergoes a partial change; and such cases are usually attended with a great offensiveness of the breath, along with dysphagia, and not unfrequently with a power of rumination.

### Institute of France.

### I. Academy of Sciences.

*Séances in July.*

#### Mortality in Armies.

According to the reports of M. Morozzo, one of the late presidents of the Academy of Turin, the mortality in the Piedmontese army, during peace, was very considerably greater than in the same number of men in civil life. From the year 1775 to 1791 the mortality among the infantry regiments was at least three times as high as among any other set or class of men residing in the same district.

M. Chateauneuf has undertaken similar inquiries into the state of the health of the French armies, and the results of his researches generally agree with those obtained by M. Morozzo. He calculated the aggregate mass of the military establishment, (exclusive of officers, cavalry, artillery, engineers, the gendarmerie, and household troops) from the year 1820 to 1826, at 803,231 men. From this number he deducted the colonial regiments, and the men discharged from the army of France in Spain upon the civil hospitals: among these last mentioned the number of deaths was very considerable. These retrenchments reduce the total to 718,994. Now during the above period the mortality amounted to 14,112—a proportion of 1.96 in 100; but this mortality does not appear to be uniform in the different sections or departments of the troops. M. C. has therefore given in the following particulars:—Non-commissioned officers, 24,370; number of deaths, 266. Drummers, 3,910; deaths, 34. Musicians, 920; deaths, 14. Master-workmen and provosts, 370; deaths, 2. Privates, 90,230; deaths, 2,034. If these data be correct, the proportional annual mortality among the privates is at least twice as great as among the non-commissioned officers.

The causes of such a preponderance may be the following, as mentioned by M. Chateauneuf. 1. The influence of nostalgia. 2. The prevalence of the various forms of syphilis. 3. The insufficiency of the diet;—a pound and a half of bread allowed for each soldier is not enough for many vigorous men; and the animal food which they purchase with their pay does not exceed three ounces per diem. 4. The frequency of duels. From M. Chateauneuf's researches it appears that the mortality in the "bagne" at Brest is less than that among the ordinary troops.

#### Monstrosity by Diplogenesis.

Dr. Scoutteten, of Metz, communicated the particulars of a case, in which two female children were born, united by their trunks. The mother, a healthy woman, 30 years of age, was delivered on the 26th July, 1833, after a gestation not distinguished by any remarkable occurrence. One of the infants was well-formed, the other was altogether acephalous; the former was baptised in the usual manner; and up to the period when she was examined by Dr. S., (11th July, 1833,*) she continued in perfect health, sucking freely, and seeming to require more nourishment than ordinary children. She slept well, and all the functions were duly performed. The stature was 23 inches; the flesh was soft and not so firm as that of the acephalous twin; the colour of the skin was natural; the navel well formed, and the cord and placenta regular. The acephalous monster was eleven inches in length; it adhered by the lower part of the chest and the upper half of the abdomen to the corresponding parts of the other child. There was no trace of umbilicus; and it was immediately

* There must be a mistake in the dates; for we are previously told that the child was born on the 26th July; probably it ought to be 26th June.—*Editor.*

above the situation where it ought to have been that the disjunction of the two bodies took place. The lower extremities were well developed; the flesh firm, the legs and feet rather small in proportion to the thighs; the joints stiff and semi-anchylosed. The upper extremities were less developed, the right one being atrophied; and there were only four fingers on the right hand, and these were almost completely grown together. The left arm was better formed; the hand complete, and the fingers, although stiff, were capable of some motion. The vertebral column was bent considerably to the right side; it terminated abruptly on a level with the top of the shoulders; the cervical vertebræ, with the exception of the seventh perhaps, were awanting. At the point of termination, was a distinct, rounded scar, about four inches in extent. There was no anus; but, with this exception, the inferior part of the trunk was normal. There had been no apparent voluntary motion in any part; but it frequently happened that the other child "jouait avec les membres de sa sœur." Although the muscles of animal life were impotent, those of organic life were capable of action; for the bladder contracted and expelled some urine with considerable force. This discharge took place at different times from the two children. We are not informed when this heteradelphous monster (to use St. Hilaire's expression) died; but, as the preceding account was laid before the Academy on the 30th July, and M. Serres read a report of the dissection on the 12th August, it was probably a few days anterior to the last date.

The vascular communication between the parasitic acephalite and the other child was principally by two arteries; one of these was a continuation of the internal mammary, and gave origin to the brachial vessels; the other was sent off from the cœliac trunk, and supplied the pelvis and lower extremities. [This description is disgracefully incomplete.] M. Scoutetten is mistaken when he states that this double monster is the only one which has lived. Several others are on record; one born in the Faubourg St. Antoine, lived for some years, and the parents used to carry it about the country for public shew.

*Séances in August.*

### LITHOTRITY.

M. Leroy D'Etiolles communicated the results of fifteen patients on whom he had performed the operation of crushing the stone by percussion and pressure. Complete and very speedy success was obtained in fourteen of these cases: a success infinitely greater than that of M. Civiale at the Hôpital Necker, where, during two years, only 27 cures in 43 cases have been effected. The great superiority of the hammering and crushing lithotritic instruments over those which act by successive perforations is thus incontrovertible; and the testimony of M. L. is the more valuable, seeing that he himself contributed not a little to improve the original operation by means of these last.

### BAREGINE, THE FATTY MATTER FOUND IN MINERAL WATERS.

This is a highly azotized substance which is found in sulphureous thermal waters: when very pure it resembles considerably calf-foot jelly, has no colour, is inodorous, and remains unchanged in the air. It is very sparingly soluble in water, requiring at least a hundred-thousand times its weight; and yet in this extremely small proportion it communicates a certain degree of viscidity to it. When dried and submitted to distillation, it yields an oily matter, some ammonia, and a large quantity of carbon difficult of incineration. When a thermal water is exposed to the air, the baregine appears, not under the form of a uniform gelatinous matter, but in long white filaments, which become of a greenish hue, when the mineral water meets a stream of common water. According to M. Longchamp, the characters of baregine resemble a good deal those of fibrine; like it, it is almost insoluble in water; very little soluble in acids and alcalis at the ordinary temperatures, and when treated with boiling nitric acid yields oxalic acid, and the bitter product of Welther.

### Calculous Affections.

The conclusion which M. Civiale has deduced from his researches, embracing 1881 cases observed in different localities, are the following. 1st. The number of children affected with calculous disorders is greater than is usually supposed. Out of the 1881 cases, 1126 occurred in patients under 14 years of age. 2. The number of patients who have calculi in the urethra, is also much more considerable than is generally stated. 3. In many situations the difficulty of procuring good surgical assistance, and the dread of undergoing the cutting operation induce many to conceal their malady ; and thus not a few die without the presence of a calculus having been detected. 4. The mortality, after lithotomy, is greater than we are taught by surgical writers to believe; thus, out of 1644 operations there were 1276 cures, and 234 deaths. Now, if we recollect, that nearly two-thirds of the patients operated upon were children, in whom the chances of success are at least two-fold, we are forced to the conclusion, that the data furnished by most modern authors are extremely inaccurate.

In fine, says M. Civiale, (when alluding to the success which some surgeons have boasted to have obtained in their practice), experience has proved that the operation of lithotomy may be performed, in a considerable number of cases, without the sacrifice of one life; while, in other cases and under other circumstances, almost every patient has died.

The following statistics, relating to calculous diseases, are given by M. Civiale.

In Nice and its neighbourhood, embracing a population of 32,000 inhabitants, there occurred only five cases in the space of ten years. Two were in children, and three in adults—three cured, one died, and one had incurable fistula remaining.

In Geneva and its territory, with a population of 200,000 inhabitants, 20 cases occurred in seven years: 10 of these were in children, 7 in adults, and 3 in old men. Out of 17 operations, 12 were successful, and 5 fatal.

In Malta and the adjacent islands, having a population of 180,000, only 4 cases occurred—the operation was performed 3 times, and was successful in 2.

In Malaga, with a population of 60,000 inhabitants, only 6 cases occurred. The operation of lithotomy was performed in all these, and appears to have been singularly unfortunate, for one died, and the other five had fistulæ remaining.

In Naples, out of 308 cases of calculus admitted into the hospitals, and operated upon, 261 were cured, and 47 died; 129 of these cases occurred in children, 148 in adults and old men.

In the Lombardo-Venetian territory, embracing a population of 60,000 inhabitants, we have the details of 30 calculous cases, 4 of which occurred in women, and 23 in male children, and 3 in adults. The lateral operation, after the method of Dubois, was performed in all, and there was only one death, and one case of remaining fistula.

In Venice itself, the number of calculous cases, during a space of 10 years, admitted into the provincial hospital, amounts to 68 : 4 of these were in females—out of the remaining 64, 44 were in children, 19 in adults, and 5 in old men. The operation was performed 63 times, either with Hawkins' gorget, or with Frere Côme's lithotome caché ; 19 of the patients died, and 44 were cured.

From the province of Brescia, having a population of 329,000 inhabitants, 175 cases of calculus are detailed—147 of these were in children, and 28 in adults. The operation of lithotomy was performed 172 times; in 108 of the cases by the lateral method—in 45, by the recto-vesical—in 4, by the Celsian —in 10 by uretrotomy, and in one case by the high, or supra-pubal incision.

In Milan and its environs, with a population of 538,173 inhabitants, 127 cases occurred in 10 years—91 were cured, and 36 died.

At Vienna, the surgical school has admitted, in the course of ten years, 70 cases of calculus. In 63 the operation was performed, and was successful in 48.

*Séances in September.*

STATISTICS—MORTALITY.

M. Moreau de Jonnes, stated some interesting results of his inquiries. It appears that the difference in the mortality of different countries is much greater, than the difference in the number of births—the maximum of the former exceeding the minimum nearly threefold [22, 59], whereas the maximum of reproduction is not higher than double the minimum. The mortality in the Roman states, in the old Venetian territories, in Greece and Turkey amounts to 1 in 30,—in the Low Countries, in France and in Prussia, 1 in 39—in Switzerland, Austria, Spain and Portugal, 1 in 40—in Russia and Poland, 1 in 44—in Germany, Denmark and Sweden, 1 in 45—in Norway, 1 in 48—in Ireland, 1 in 53—in England, 1 in 58—and in Scotland 1 in 59. The two leading causes which influence the population of a country, are its climate, and the degree of its civilization. A cold climate is certainly more favorable to life than a warm one; and if we examine the rate of mortality in countries within the Torrid Zone, it is much higher than in one of more temperature; thus in Batavia, it amounts to 1 in 26 —in Trinidad, 1 in 27—in Martinique, 1 in 28—at Bombay, 1 in 20—at Havannah, 1 in 33. Heberden rated the mortality in the island of Madeira, at 1 in 50. To illustrate the beneficial effects of civilization, the following details are very interesting. In Sweden, from the year 1754 to 1763, the mortality was 1 in 34;—from 1820 to 1825, it was only 1 in 45. In Great Britain, from 1787 to 1789, it was 1 in 43. In France, in 1776, it was 1 in 25½.

The medium of mortality throughout Europe was calculated many years ago at 1 in 36.

QUARANTINE ESTABLISHMENTS.

That indefatigable anti-contagionist, Dr. Chervin, engaged the attention of the Academy with a learned refutation of the reasonings of M. Segur-Dupeyron, the Secretary of the Council of Health, in favour of continuing the present quarantine regulations. The first position of M. D. is, that the countries of Europe, which are known to be most frequently in contact with pestilential diseases, are those, where the doctrine of contagion numbers most partizans. M. Chervin disputes this statement; it is especially inapplicable to America; for at New Orleans, one of the places, which above almost every other, has suffered from the yellow fever, quarantines have been abolished since the year 1825. Even in Spain, where liberty of discussion is prevented, the quarantine enactments are less severe than at our own Marseilles:—The administration of France is decidedly more "contagionist" than that of Spain, and has been the chief cause of retarding those improvements, which the sanitary system of Europe so much demands. England and Holland would have most certainly made the experiment of a greater toleration, had France not mischievously thrown obstacles in their way. The very expense of supporting a large quarantine establishment is immense; the goods too are often much damaged; vessels are destroyed by being obliged to keep in bad anchorages, and sailors often refuse to go in ships, when exposed to the annoyance of imprisonment for 30, or 60 days. The commerce of France has sustained great injury from the strict quarantines imposed, while that of other nations relieved from such vexatious hindrances has proportionally prospered. In England, vessels coming from any part of America are admitted; with us, however they are not; and the Egyptian and Levantine trade is much less fettered in the one country than in the other. M. Chervin calculates that one twelfth of the French shipping is constantly locked up in quarantine.

---

### II. Academy of Medicine.

*Séances in August.*

### Pericarditis induced by the Presence of a Needle in the Right Ventricle of the Heart.

Dr. Renauldin and M. Boujet, of the Hospital Beaujon, communicated the particulars of this very curious case. A man, aged 63, had come from the country to Paris, with the view of settling some of his affairs. It was soon discovered that he laboured under suicidal mania; he wrote a letter, that he was to die in five or six days; and he kept his bed, without taking any nourishment, excepting a little coloured water. One night, be fastened a cord round his neck, and when he was thus found in the morning, he swore that some savages had tried to strangle him.

On being taken to the Hospital Beaujon, he complained of an asthma and oppression at the chest. Percussion elicited a duller sound than natural, at the right anterior part of the chest, and the respiratory murmur was found to be wanting there. The respirations were 27 in the minute, the pulse 129, full, and hard. He could lie on either side; for a few days he found relief from the means which were employed; but upon the 5th day, after his admission, the dyspnœa and oppression increased exceedingly, and while attempting to speak, he suddenly fell back and died.

*Dissection.* The pericardium was distended with two pints of fluid; the bag was much thickened by inflammation, and its inner surface was granulated, and lined with layers of albumen. The heart, at its apex, had contracted an adhesion to it. On cutting open the right ventricle, a needle, three inches at least long, was found fairly imbedded within its walls; its direction was from before backwards, and from above downwards; and it appeared to have penetrated into the cavity of the ventricle. Probably it had been introduced, through one of the intercostal spaces; but no trace of any cicatrix, however small, could be found; how long it had been there, there were no means of discovering; the monomania had existed for several weeks. Perhaps this state of mind was the cause, why the patient did not complain of any uneasiness, or pain in the part.

### Amputation of the Leg, effected by a Ligature.

A man aged 24, while engaged in field labour, was bit by a viper, at the lower and back part of the left leg. According to the usual practice of his country (Lithuania), he fastened a cord firmly around the limb above the wound, and about four inches beneath the patella, and this girth he could tighten still more, by twisting it with a short stick. He allowed it to remain on his leg from the 10th June to the 23rd July, on which day the mortified limb dropt off; a considerable hæmorrhage supervened; the wound was oblique, and the stump was of a conical shape, the anterior crest of the tibia being the most projecting part. The skin had already commenced the cicatrizing process, and the fleshy surface was suppurating freely, and covered with healthy granulations—the divided bones felt somewhat rough to the finger, and the fibula had become dead in almost its whole length.

It was referred to MM. Sanson and Adelon, who gave it as their opinion that amputation should have been practised, in order that a more secure and convenient stump might be obtained. A case altogether similar occurred in the person of a young Greek, during the war of the Morea in 1826. He had been bit by a serpent, and applied the ligature himself. He was under the care of the surgeon-major, M. Petitot.

### Cancer of the Heart and of the Kidneys.

A washerwoman, aged 65, was admitted into the Hospital Beaujon, in a state of great debility and emaciation. On the left side of the abdomen, and just below the edge of the ribs, there was a hard, irregular, and painful tumor, which was supposed to arise from an enlarged spleen. Her health had been failing for two years, with loss of appetite and sleep, and tendency to diarrhœa. There was no disturbance of the circulation, or of the urinary secretion; at least, if there was any, it must have been inconsiderable, as it did not arrest the attention, either of the patient or of her physician. She was relieved by soothing applications, and by frequent doses of opium, and thus lingered out a tortured existence (for the pain was excruciating) for three months in the hospital.

*Dissection.* In the right ventricle of the heart, a carcinomatous tumor, as big as a walnut, was found. Its surface was irregular, with numerous warty excrescences, like those we see in syphilis. The spleen was not diseased in structure, but much shrivelled in size; the left kidney was greatly enlarged, and presented the true cancerous degeneration throughout its substance. Two small carcinomatous tumors were found in the right kidney and in the uterus.

### HERMAPHRODISM.

M. Castel commences his memoir by stating that, up to the present time, not one case of real and genuine hermaphrodism (by which is meant the co-existence of both sexes, and the consequent simultaneous capability of impregnation and of conception) has ever been discovered, either in man, or in any of the higher animals. Not only has no such instance ever been found, but M. Castel affirms that it never will be found at any future time. Nature, although omnipotent and all-creating, never generates absurdities or incongruities, nor does she ever work, only for the display of power. There is a universal harmony throughout her countless operations; and although seemingly capricious and sportively fickle, 'tis only "seemingly," for, in the pride of our ignorance, we are unable to follow her.

If we contemplate the differences of the two sexes, in the more perfect animals, we shall find that they are not merely physical or structural, the one being distinguished by testicles and penis, the other by ovaries and womb: the whole organism of each suffers and sympathises with their characteristic peculiarities, and their feelings, and instincts, and dispositions are curiously modified, and become, as it were, typical of their different frames.

If this be true, as a general remark applied to the higher animals, how much more striking is it, if we contemplate the two sexes of the human species. The masculine vigour of the one cannot possibly be associated, in the same being, with the feminine tenderness of the other; yet such a strange co-existence must be present, in any case of absolute hermaphrodism. Probably no animal which is capable of a will, in the act of re-production, has ever been, or can ever be, truly hermaphroditic. The simply-organized beings, which are low in the scale of animal life, and which are possessed of the male and female generative organs, have but an indistinct nervous system, and the act of reproduction may be considered as a mere living impulse—an unconscious and involuntary function, scarce in any way different from the impregnation of the germen of a plant by the pollen, which the anther scatters on the pistil. As a general position, which we expect to be completely borne out by the facts which are already known, or which may hereafter be discovered, it may be affirmed that, in proportion as instinct and will exercise a greater or less influence on an animal, in re-producing its like, so are the chances of hermaphrodism, in their species, diminished or increased. Again, the more that the genital organs, in any tribe of animals, differ in their character and structure from the other excretory and secretory organs of the body, the less is the probability of genuine hermaphrodism ever occurring in it.

M. Bouillaud expressed his wish that the Academy should set apart a particular day for the discussion of the curious and intricate subject of hermaphrodism.

M. Adelon deemed this suggestion quite unnecessary, as the number of cases hitherto minutely examined and well authenticated, was too small for any legitimate generalization.

The pretended human hermaphrodism, he stated, is only a monstrosity of the genital organs, and never a co-existence of these parts belonging to, and characteristic of, either sex in one being. Now all the genital monstrosities may be reduced to four divisions. —1. Those of males, whose organs in some degree resemble the female sexual apparatus.—2. Those of females whose organs resemble the male apparatus.—3. When the sexual organs are so rudimentary, that we find difficulty in determining the sex of the individual,—and, 4. When there are actually present one or more of the distinctive organs of the two sexes in the same person, even although these organs are far from being perfect and complete, or from being capable both of impregnation and of conception.

When, therefore, we talk of hermaphrodites being either male, female, neutral, or mixed, the language is more convenient than it is correct. As regards the cause of hermaphrodism, in the limited acceptation of the term, the probability is that, in all cases, it is dependent upon either an arrest of development in fœtal life, or in a positive disease of the fœtus while in utero. M. Breschet followed M. Adelon, and took nearly the same view of the subject. Many of the reported cases of the mixed hermaphrodism are quite apocryphal, and he alluded to one preparation in wax, at the museum of the Faculty of Paris, which the artist has made much more wonderful than Nature had done before him. When difficulty is found in determining the sex of an infant at birth, the civil magistrate of the place may inscribe upon the registry, "sexe non determiné."

### The Advantages of turning the Fœtus by the Head rather than by the Feet.

Up to the end of the 16th century, the only mode of turning ever practised was by bringing down the head first; and we find this conduct recommended, not only in such cases as are admitted at the present day to require artificial delivery, but even in common pelvic and feet presentations. Soon after the above-mentioned date, turning by the feet was first proposed, but it was not until the commencement of the 18th century that the practice was generally followed. One of the professors of the School of Strasburg resisted this innovation, strongly maintaining the superiority of the old regime; and his advice was approved of by many of the German practitioners. To justify this preference, it was asserted that when the head presented first, the compression caused by the os uteri is not sufficient to injure the encephalic contents, and moreover, the communicant circulation between mother and child remains unobstructed; whereas in presentations of the lower extremities, the thoracic and abdominal viscera are exposed to a dangerous compression, and the fluids are driven back upon the head, thus causing frequently a fatal cerebral congestion. In confirmation of the truth of this statement, we are told that only one child in twenty delivered by the head is still-born; whereas, the proportion is one to five in feet presentations. In conclusion, it is alleged that whenever the fœtus is movable within the uterus, it is quite as easy to effect the turning by the head as by the feet.

M. Dubois dissented from the above arguments. He contended that the described dangers of any compression on the abdomen and thorax were most unnecessarily exaggerated, and instanced two cases wherein the shoulder presented along with the head, and yet the children were delivered without any contusion of the thoracic and of the abdominal viscera.

The dread too of the retropulsion of the blood upon the head was an offspring of fancy rather than a result of experience; he did not agree with them in their belief that the os uteri exercised such a constrictive pressure as was alleged; the parts of the fœtus which have already escaped from the uterus are subjected to a less degree of pressure than those still contained within its cavity; and hence we can readily explain why the blood should be driven to and accumulated in the former. Do we not observe that when an arm is born first, the member frequently becomes much swollen ? now this swelling arises from the pressure being less upon the arm than upon the rest of the body. True it may be, that in many children who die after feet-presentation, visceral congestions are not unfrequently discovered; but the cause of these is the compression of the umbilical cord, and not the retropulsion of the fluids which M. Flamant believed to take place.

The compression of the cord is a necessary danger attending all births by the feet, and indeed it constitutes a very serious objection to the process of turning; the child is very often asphyxiated, and in such a case we find upon dissection the same phenomena which are observed after drowning or hanging, viz: an apoplectic plethora within the head, great congestion in the veins of the cerebrum and other viscera.

The calculations which have been adduced to prove the greater safety of turning by the head than by the feet, are not strictly correct, as will appear from the following statement of M. Dubois.

In all such calculations, to ascertain the comparative mortality of the different modes of delivery, we must be careful to exclude from our tables all cases wherein the child has died before actual accouchement has commenced; or wherein the labour has been premature and the child may be therefore not well capable of independent life. Now the new tables which have been recently formed at the Maternité of Paris, on these principles, shew, " that from the 1st of June, 1829, to the 1st of June, 1833, 10724 children have been born at the hospital; of these, 10262 were born by the head, 391 by the lower extremity, 59 by the trunk, and—30 by the face; of the 10262, 9867 were at the full period of gestation, and 395 were not. The 9867 may be reduced to 9837, because, in 30 of the cases the fœtus was known to be dead before delivery commenced, and the 395 premature cases may be reduced to 278 ; for in 83 the fœtus had been dead for some time, and in 34 it was too imperfectly developed for the maintenance of independent life.

Of the 9837 deliveries by the head, at the full time, 191 were born dead; the proportion is therefore one in 51 or 52 ; and of the 278 prematurely born, 48 were born dead, or one in every 5 or 6. Of the 391 deliveries by the lower extremity, 238 were at the full term, and 153 before the term ; from the first number we must deduct 7, who were dead before labour began; and out of the remaining 231, 21 were born dead; a proportion of one to eleven. From the 153 we must deduct 63, in which the child had evidently died during pregnancy, and 30, in which it was too young for independent life ; and out of the remaining 60, 10 were born dead; or one in six. From these calculations it appears among other results, that the fœtus at the full period can endure the "*fatigues of accouchement*" with much greater safety than when born at an earlier period, whether they are delivered by the head or not. M. Dubois draws our attention to the important difference in the results by the previous deduction of all the cases in which the fœtus either had been dead for some time before labour, or was incapable of life when delivered. Thus had we enumerated these cases among the mortality in the 10262 head presentations, we should have had 386 deaths, or one in 25 ; whereas we have fixed it above at one in 51 : and in the 391 feet-presentations the deaths would have amounted to 134, or nearly one in two, instead of one in eleven. With regard to the comparative advantages in practice of turning by the head, M. D. admits that in some cases the operation is not only quite possible, [Mad. Lachapelle was wrong in denying this,] but also abundantly easy. He has himself performed it twice when the shoulder presented; but the operation is much more difficult than that of turning by the feet, and should the liquor amnii have copiously escaped, or should the uterus be firmly contracted around the child, the manœuvre is almost impracticable. In the 59 trunk presentations, two were delivered by means of turning by the head; in a third case the expulsion of a putrid fœtus took place by the shoulder; and in the remaining 56 the child was brought down by the feet. Out of the whole number 59, in 25 only did the child survive; but M. Dubois is of opinion that a still smaller number would have been saved had turning by the head been tried in all.

### Cholera at New Orleans.

It appears from an able memoir of Dr. Halphen of New Orleans, that the cholera first broke out, during the existence of a most severe epidemic of yellow fever. In the month of September of last year, a good many cases of the yellow fever were observed; but it was not until the middle of the following month, that it was declared epidemic in the place; and about the same time, the new pestilence added its horrors. The character of the former, was as formidable, as it had ever been known on any former year; and all the cases required general bleeding, at the beginning and the strictest antiphlogistic regimen. It seems that this depleting treatment favoured the development of the cholera. In the practice of Dr. H. eight, or ten cases of cholera, occurred in patients actually labouring under yellow fever; but in proportion as the one (the cholera) prevailed, the other became less intense. On the 12th of November, a cold northerly wind sprung up, and in three days, the pestilence had entirely vanished.

Dr. H. gives it as his opinion that the cholera was brought to New Orleans from Saint Louis, by the Constitution steam-boat. The remedy which Dr. H. found by far the most efficacious, was a combination of sulphate of quinine with 'thridace;' three grains of the former and one grain of the latter, every fifteen, or twenty minutes, until re-action was produced. Enemas with the same were also given.

### Tarentism.

This hypochondriacal and convulsive affection, is caused, by the bite of the Tarentula. It is most common in the province of Otranto, at the south east side of Italy. The part bitten becomes red and inflamed; and the swelling extends for some distance all round. Some hours afterwards the patient becomes sorrowful, desponding and silent; feels much anxiety and tightness across the chest, and is distressed with vertigo, nausea and vomiting; he gradually sinks into a state of dulness and apathy; and the mere remembrance of his wretchedness, the return of summer heat, or seeing another person afflicted in the same way, as he is himself, brings on severe paroxysms of hypochondriasis. The treatment consists in making the patients dance to the sound of the violin, or bagpipe; and sure enough it is often successful; whether from the copious perspiration induced, or merely from the hilarity acting on the imagination, we must leave to others to determine. The theriaca and ammonia are at the same time to be given inwardly, and the external use of the ammoniacal soap is also recommended. Dr. Renzi of Naples, the reporter, positively denies the truth of the opinion that Tarentisme, is a form of hypochondriasis, depending solely upon the climate; the only true cause, according to him, being the bite of the Tarentula. He adduces two illustrative cases; one of which occurred in an infant of three months, and the other, in a peasant who was bit during sleep. In both a cure was effected by music.

The Academy was willing to give Dr. Renzi credit, for his industry in collecting the facts; but not for the authenticity of the details. Tarentism is in all senses an imaginary disease.

### *Séances in September.*

### Peritoneal Extra-uterine Pregnancy.

The body of a woman, 78 years of age, was brought to the anatomical theatre of Geneva, and on dissection the following curious anomaly was found.

A tumor, of a hard cartilaginous consistence, occupied the right side of the pelvic cavity; it adhered intimately to the bladder, uterus, and vagina, but did not communicate with any of them. On cutting it open, a mummified fœtus, of about three months, was discovered within. The most minute examination could not ascertain how the fœtus had originally been detached, whether from the ovary or fallopian tube; or whether it had made its escape by a rent from the uterus or vagina. The woman was the mother of three children, and had enjoyed good health, ultimately sinking under the effects of old age. The fœtus had been lodging in its cyst for upwards of thirty years; when taken out, it was found to be encrusted over with a layer of phosphate of lime. M. Cloquet, who read the memoir of M. Majore of Geneva, regarded the present case as an example of true peritoneal pregnancy, the existence of which in the human subject has been so often contested. M. M. Breschet and Beclard, after having carefully examined the reports of all the cases of supposed peritoneal pregnancy on record, were still obliged to refuse their credit to the authenticity of such an occurrence.*

M. Cloquet, in reply, alluded to the admitted occurrence of this anomaly in some of the lower animals; for example, in cats, in which he had seen fœtuses developed within encysted sacs, and these sacs adhering to the peritoneum by means of blood-vessels. M. Velpeau supported these latter views, and adduced two observations from his own experience,—the fœtuses, about three months advanced, had no connexion whatever with the ovary, fallopian, or uterus.

M. Capuron and M. Esquirol relate two other analogous cases. In that detailed by the latter, the woman was 68 years of age. M. Moreau stated that he had once examined a rabbit, in whose abdomen were several fœtuses floating about and quite detached. It is to be remembered that in all such cases the fœtuses are never complete, seldom having reached beyond the early stages of gestation. In the possibility of actual peritoneal pregnancy M. Lisfranc also coincided. The testimony of Professor Lallemand of Montpelier is favorable to the same side of the question;—he has mentioned in his inaugural thesis the case of a woman, who, being affrighted during the act of coition, was immediately seized with a violent pain on one side of the belly. Eight months afterwards she died of extra-uterine gestation; and when opened, the fœtus was found in the situation of her sufferings.

M. Breschet alleged however that in Lallemand's case the fœtus was found between the ovary and the Fallopian tube, and that therefore it ought not to be admitted as one of genuine abdominal pregnancy.

* The explanation which they gave was, that during the fœtal life of the patient herself the germ of a twin child had become included within the body, just in the same way as had happened in the case of the young Bissieu, reported by Dupuytren, and recorded in the bulletin of the faculty.

### Eclampsia of Young Infants.

M. Duges explained that this convulsive disease of infancy always depends upon some irritation of the encephalon; that it sometimes is followed by an apoplectic or asphyctic state, and at other times is consecutive to it.* Often while one side of the body is convulsed the other side is paralysed. There is also a strong analogy, if not an absolute identity between infantile eclampsia and the tetanos, which has been improperly constituted a peculiar disease, and said to be exclusively belonging to the West Indies. M. Duges has repeatedly witnessed cases of genuine general as well as partial tetanos in infants at home. There are therefore, according to him, three species of eclampsia, viz. the epileptic, the apoplectic, and the tetanic.

* It not unfrequently happens that the symptoms of eclampsia, of apoplexy, and of asphyxia, are so blended and confused together in new-born children, that it is extremely difficult to distinguish the exciting from the excited disease, or to decide whether they are all of simultaneous occurrence. Very soon after birth, probably the apoplexy generally precedes the eclampsia; and in children more advanced, the latter seems to induce the former.

